# Uncovering the roles of microRNAs/lncRNAs in characterising breast cancer subtypes and prognosis

**DOI:** 10.1186/s12859-021-04215-3

**Published:** 2021-06-04

**Authors:** Xiaomei Li, Buu Truong, Taosheng Xu, Lin Liu, Jiuyong Li, Thuc D. Le

**Affiliations:** 1 UniSA STEM, University of South Australia, Adelaide, Australia; 2 School of Life Sciences, University of Science and Technology, Hefei, China; 3 Centre for Cancer Biology, University of South Australia, Adelaide, Australia

**Keywords:** miRNA, lncRNA, Breast cancer, Subtype discovery, Cancer prognosis, Method comparison

## Abstract

**Background:**

Accurate prognosis and identification of cancer subtypes at molecular level are important steps towards effective and personalised treatments of breast cancer. To this end, many computational methods have been developed to use gene (mRNA) expression data for breast cancer subtyping and prognosis. Meanwhile, microRNAs (miRNAs) and long non-coding RNAs (lncRNAs) have been extensively studied in the last 2 decades and their associations with breast cancer subtypes and prognosis have been evidenced. However, it is not clear whether using miRNA and/or lncRNA expression data helps improve the performance of gene expression based subtyping and prognosis methods, and this raises challenges as to how and when to use these data and methods in practice.

**Results:**

In this paper, we conduct a comparative study of 35 methods, including 12 breast cancer subtyping methods and 23 breast cancer prognosis methods, on a collection of 19 independent breast cancer datasets. We aim to uncover the roles of miRNAs and lncRNAs in breast cancer subtyping and prognosis from the systematic comparison. In addition, we created an R package, CancerSubtypesPrognosis, including all the 35 methods to facilitate the reproducibility of the methods and streamline the evaluation.

**Conclusions:**

The experimental results show that integrating miRNA expression data helps improve the performance of the mRNA-based cancer subtyping methods. However, miRNA signatures are not as good as mRNA signatures for breast cancer prognosis. In general, lncRNA expression data does not help improve the mRNA-based methods in both cancer subtyping and cancer prognosis. These results suggest that the prognostic roles of miRNA/lncRNA signatures in the improvement of breast cancer prognosis needs to be further verified.

**Supplementary Information:**

The online version contains supplementary material available at 10.1186/s12859-021-04215-3.

## Background

Breast cancer accounted for 24.2% of all new cancer cases in women in 185 countries in 2018, being the leading cause of cancer death for women at the same time [1]. In order to improve the survival outcome of patients with breast cancer, it is urgent to develop and use accurate diagnostic and prognostic tools to assist clinicians and patients in therapeutic decision-making.

Breast cancer is an extremely complex disease with different subtypes and heterogeneous treatment responses. Traditional breast cancer diagnosis and prognosis are based on clinicopathological variables, such as histologic tumour grade, lymph node status, and tumour size [2–4]. However, these methods alone are not sufficient to guide the choice of effective treatment because breast cancer is a disease that is not only pathologically and clinically diverse, but also biologically different [5]. With the advent of new sequencing technologies, researchers have extensively used genomic data to identify molecular subtypes of breast cancer [6–15] and gene signatures for prognosis [15–23]. These methods have been successful in stratifying patients into several subtypes, each of them with distinct biological and clinical characteristics. The molecular-based subtypes and the gene signatures for prognosis are being translated into clinical practice in recent years [15–17, 23].

There have been some works on reviewing breast cancer subtyping or prognosis methods and some software packages have been developed for breast cancer subtyping or prognosis. Russnes et al. [24] reviewed breast cancer classification and stratification methods and compared two specific methods, PAM50 [15] and IntClust [9], which result in so-called intrinsic subtypes and integrative clusters, respectively. The comparison results showed that the integrative clusters captured the intrinsic subtypes and some novel breast cancer subtypes that had distinct copy number variation patterns. The CancerSubtypes package [25] provided a framework for identifying cancer subtypes using multi-omic data from the TCGA project. The genefu package [26] provided computational methods for breast cancer subtyping and prognosis that were based on gene signatures. Yu et al. [27] summarized current multi-gene signatures into three categories: (1) biological pathway-based prognosis signatures, (2) the first generation prognosis signatures and (3) the second generation prognosis signatures. Similarly, several works [28–34] reviewed the relative literature, without conducting comparative studies. However, there are two major limitations to these reviews. First, previous reviews covered either breast cancer subtyping methods or prognosis methods, but not both (with the exception of genefu which covers few gene-based methods). Although breast cancer subtyping and prognosis are in different sub-areas, both of them could lead to advance personalized treatment of breast cancer patients and to improve their survival outcomes. Moreover, breast cancer subtyping can help with prognosis by providing signatures or other prognostic information. An example can be found in [27], where the author confirmed subtype-specific signatures outperformed other gene signatures in the risk stratification of the corresponding cohorts. Second, there is no work that systematically analyzed the breast cancer subtyping and prognosis based on multiple levels of transcriptomic data, specifically, mRNA, miRNA and lncRNA expression data.

However, research into miRNAs/lncRNAs and their roles in cancers have been extensive in the last couple of decades and substantial works have shown the significant roles of miRNAs/lncRNAs in cancer development and progression [35–37]. Recent works have also utilised miRNA expression data for breast cancer subtyping and prognosis [11, 38]. An increasing number of breast cancer prognosis methods have been developed to select prognostic signatures from human lncRNAs and trained survival models based on the selected signatures [39–41]. However, it is not clear whether miRNA/lncRNA data is more effective than other omic data for subtyping and prognosis and whether it is useful to incorporate miRNA/lncRNA data with other omic data.

To address this question, in this paper we evaluate 35 breast cancer subtyping and prognosis methods through empirical study. Nineteen breast cancer datasets were collected, five of them have matched miRNA-mRNA expression data, seven of them have matched lncRNA-mRNA expression data and one of the datasets contains matched mRNA-miRNA-lncRNA expression data. For each of the multi-omic cancer subtyping methods, we evaluate its performance in different scenarios, when using single omic data and combinations of multiple omics data. By doing this, we can compare and observe the cases with and without miRNAs/lncRNAs on the same cohorts of breast cancer patients. The 23 breast cancer prognosis methods vary from gene based methods, miRNA based methods, and lncRNA based methods. We evaluate the breast cancer prognosis methods based on their applicable data in the 19 datasets.

All these comparisons and analyses allow uncovering the roles of miRNAs/lncRNAs in characterising breast cancer subtyping and prognosis. Besides, through such a comparative study, we present a set of practical recommendations on the use of the existing methods and the development of new computational methods for breast cancer subtyping and prognosis. We make all the processed data and codes (as an R package) available to facilitate the study and the development of breast cancer subtyping and prognosis methods.

## Materials and methods

### Datasets

**Table 1:** A summary of the datasets

Datasets	#Features	#Patients	Data types	Platforms	Source
TCGA753	18104	753	miRNA, mRNA	Illumina Genome Analyzer miRNA Sequencing, Illumina HiSeq 2000 RNA Sequencing V2	[42]
METABRIC	25191	1283	miRNA, mRNA	Agilent ncRNA 60k, Illumina HT 12	[9]
UK	22172	207	miRNA, mRNA	Illumina Human v1 MicroRNA expression beadchip, Illumina humanRef-8 v1 expression beadchip	[43]
HEL	25946	115	miRNA, mRNA	Illumina Human v2 MicroRNA expression beadchip, Illumina HumanHT-12 V3 expression beadchip	[44, 45]
GSE19783	20085	99	miRNA, mRNA	Agilent-019118 Human miRNA Microarray 2 G4470B, Agilent-014850 Whole Human Genome Microarray 4x44K G4112F	[46]
TCGA500	31729	500	lncRNA, miRNA, mRNA	Illumina Genome Analyzer miRNA Sequencing, Illumina HiSeq 2000 RNA Sequencing V2	[42]
GSE12276	54675	204	lncRNA, mRNA	Affymetrix Human Genome U133 Plus 2	[47]
GSE19615	54675	115	lncRNA, mRNA	Affymetrix Human Genome U133 Plus 2	[48]
GSE20685	54675	88	lncRNA, mRNA	Affymetrix Human Genome U133 Plus 2	[49]
GSE20711	54675	252	lncRNA, mRNA	Affymetrix Human Genome U133 Plus 2	[50]
GSE21653	54675	77	lncRNA, mRNA	Affymetrix Human Genome U133 Plus 2	[51]
GSE42568	54675	327	lncRNA, mRNA	Affymetrix Human Genome U133 Plus 2	[52]
GSE9195	54675	77	lncRNA, mRNA	Affymetrix Human Genome U133 Plus 2	[22]
TRANSBIG	22283	198	mRNA	Affymetrix Human Genome U133A	[53]
UNT	44928	137	mRNA	Affymetrix Human Genome U133A/B	[18]
UPP	44928	251	mRNA	Affymetrix Human Genome U133A/B	[54]
MAINZ	22283	200	mRNA	Affymetrix Human Genome U133A	[55]
NKI	24481	337	mRNA	Agilent Human oligo microarrays	[16, 56]
GSE6532	44928	414	mRNA	Affymetrix Human Genome U133A/B/Plus 2	[22]

*#Features* number of features in the cohort, *#Patients* number of patients in the cohort

For a systematic analysis of breast cancer subtyping and prognosis on mRNA, miRNA, and lncRNA data, we use 19 genome-wide expression datasets containing 5134 breast cancer patients from different repositories (details in Table 1). The mRNA and miRNA expression data from TCGA breast cancer datasets (TCGA753 and TCGA500) were downloaded from FireBrowse^1^ (data version 2016_01_28). The METABRIC breast cancer dataset was a combination of the mRNA expression data (EGAS00000000083) and the miRNA expression data (EGAS00000000122) from the European Genome-phenome Archive.^2^ The MAINZ, TRANSBIG, UPP, UNT, and NKI datasets were from publicly experimental data packages in Bioconductor.^3^ The remaining datasets were all downloaded from the Gene Expression Omnibus (GEO) database.^4^ The UK dataset was from GSE22216 for miRNA expression data and GSE22219 for gene expression data. The HEL dataset contains miRNA expression profiles from GSE43040 and gene expression profiles from GSE24450. All the datasets contain clinical outcomes, including relapse-free survival time (UPP, GSE6532, TCGA753, TCGA500, METABRIC, UK, GSE12276, GSE19615, GSE20711, GSE21653, GSE42568, and GSE9195), distant metastasis free survival time (TRANSBIG, UNT, MAINZ, NKI, and GSE20685), breast cancer death or distant metastasis free survival time (HEL) and disease-free survival time (GSE19783).

The lncRNA expression profiles of the TCGA500 cohort were acquired from The Atlas of Noncoding RNAs in Cancer (TANRIC) [57]. For the sake of comparative evaluation of breast cancer subtyping and prognostic methods using lncRNA, we applied the pipeline as previously described by Zhou et al. [39] to re-annotate GEO gene expression datasets generated by Affymetrix HG-U133 Plus 2.0 arrays. The raw microarray data of GSE12276, GSE19615, GSE20685, GSE20711, GSE21653, GSE42568, and GSE9195 were normalised using the Robust Multichip Average (RMA) algorithm [58] in the *affy* R package [59]. The probe set IDs of Affymetrix HG-U133 Plus 2.0 array were mapped to genomic locations and Ensemble IDs (Release 82, November 11, 2015) [60] based on the latest version of the NetAffx Annotation File (Release 36, Match 15, 2016). Specific probe set IDs and Ensemble IDs of lncRNAs were obtained by matching the genomic locations of probes to the chromosomal locations of lncRNAs in the GENCODE database (Release 23, March 2015).^5^ Using this pipeline, we annotated 1,938 lncRNAs from the above mentioned datasets.

### Breast cancer subtyping and prognosis methods

PAM50 [15] and IntClust [9] are two well-known gene-based breast cancer subtyping methods. PAM50 pre-defines five intrinsic cancer subtypes and eventually maps patients to one of the subtypes. IntClust stratifies patients into ten integrative breast cancer subtypes that show substantial variation in their molecular and clinical characteristics. At present, there are numerous computational methods used to identify cancer subtypes based on omics data [25, 61]. These methods can be used for both single-omic data and multi-omic data. Integrating multi-omic data has the potential to characterize human cancer at system level and further be used in treatment decisions. These methods can be classified into three categories: concatenation based methods, similarity based methods, and model based methods. The concatenation based methods concatenate multi-omic data to a single data matrix, and then utilise existing clustering methods on the integrative data. This concatenation based integration increases the dimensionality of the data and the time complexity of methods. Similarity based methods transform original multi-omic matrices into a similarity matrix. This matrix can be used by similarity-based clustering algorithms, including spectral clustering [62], PAM [63] and Dynamic Tree Cut [64]. Model based methods like iCluster family (iCluster/Plus/Bayes) [8, 65, 66] use joint statistical modelling to determine the distribution of the observed data and make effort to reduce the dimension of the data. From each category, we pick the most prominent methods on the basis of citations and publication impact (CC [6], CNMF [7], iCluster [8], and SNF [10]) and some recently developed tools (WSNF [11], SNF-CC [25], CIMLR [12], NEMO [14], PINS [13]), and intNMF [67]. These methods are not specific to breast cancer, but they are applicable to breast cancer datasets.

Since most breast cancer prognosis methods have trained linear survival models based on a similar methodology, the significant difference between these methods lies in the selection of signatures. According to the selected cancer signatures, computational methods for breast cancer prognosis can be categorised into three groups, gene-based methods [15–19, 21–23], miRNA-based methods [38, 43] and lncRNA-based methods [39–41]. Gene-based methods conduct gene marker collection and gene expression data analysis to study the relationship between gene expression profiles and clinical outcomes such as subtypes [15, 21], survival outcomes [16, 17, 19, 23], treatment responses [68], and tumour histologic grades [18]. miRNA-based methods aim to understand the role of miRNAs in either tumour-suppressive or oncogenic mechanisms in breast cancer [43]. The study in [38] investigates the matched miRNA-mRNA profiles to infer novel mixture miRNA-mRNA markers for breast cancer prognosis. Recently, it has been found that the functional dysregulation of lncRNAs contributes to cancer development. LncRNA-based methods identify lncRNA signatures involved in breast cancer metastasis-related pathways and been independent of clinical variables and subtypes [39–41]. To uncover the roles of miRNAs and lncRNAs in breast cancer prognosis, we selected 23 breast cancer prognosis methods, including 12 gene-based methods, 1 miRNA-mRNA based method, 4 miRNA-based methods, and 6 lncRNA-based methods. These methods are either widely used in breast cancer prognosis or more recently developed methods.

A detailed introduction to these breast cancer subtyping and prognosis methods is provided in Additional file 1. A summary of these methods is listed in Table 2.

**Table 2:** The computational methods included in this study

Abbreviation	# RNA	Cohort	Applicable data	Purpose	References
CC	–	BC	Multi-omics	Subtyping	[6]
CNMF	–	BC	Multi-omics	Subtyping	[7]
iCluster	–	BC	Multi-omics	Subtyping	[8]
IntClust	—	BC	mRNAs	$\hbox {Subtyping}^1$	[9]
SNF	–	BC	Multi-omics	Subtyping	[10]
SNF-CC	–	BC	Multi-omics	subtyping	[25]
WSNF	–	BC	mRNAs, miRNAs	Subtyping	[11]
CIMLR	–	BC	Multi-omics	Subtyping	[12]
PINS	–	BC	Multi-omics	Subtyping	[13]
NEMO	–	BC	Multi-omics	Subtyping	[14]
intNMF	–	BC	Multi-omics	Subtyping	[67]
PAM50	50	BC	mRNAs	$\hbox {Subtyping}^1$	[15]
rorS	50	BC	mRNAs	Prognosis	[15]
GENE70	70	ER+	mRNAs	Prognosis	[16]
OncotypeDX	21	ER+	mRNAs	Prognosis	[17]
GGI	97	ER+	mRNAs	Prognosis	[18]
Tamr13	181	ER+	mRNAs	Prognosis	[19]
AURKA	1	BC	mRNA	Prognosis	[20]
ESR1	1	BC	mRNA	Prognosis	[20]
ERBB2	1	BC	mRNA	Prognosis	[20]
GENIUS	314	BC	mRNAs	Prognosis	[21]
PIK3CAGS	278	ER+	mRNAs	Prognosis	[22]
EndoPredict	11	ER+	mRNAs	Prognosis	[23]
Ensemble	238	BC	mRNAs	Prognosis	Additional file 1
miR-21	1	BC	miRNA	Prognosis	[69–71]
miR-155	1	BC	miRNA	Prognosis	[72]
miR-210	1	BC	miRNA	Prognosis	[73, 74]
RNAmodel	37	BC	mRNAs, miRNAs	Prognosis	[38]
miRNA10	10	BC	miRNAs	Prognosis	[43]
HOTAIR	1	BC	lncRNA	Prognosis	[75]
MALAT1	1	BC	lncRNA	Prognosis	[76]
DSCAM-AS1	1	BC	lncRNA	Prognosis	[77]
lncRNA12	12	BC	lncRNAs	Prognosis	[39]
LncRNA6	6	ER+	lncRNAs	Prognosis	[40]
LncRNA5	5	BC	lncRNAs	Prognosis	[41]

Abbreviations for prognostic methods are defined in Additional file 1. # RNA, number of RNA signatures used in methods; $^1$, the method maps breast cancer patients to predefined subtypes; mRNA, messenger RNA; miRNA, microRNA; lncRNA, long non-coding RNA; BC, breast cancer; ER+, estrogen receptor-positive breast cancer

### Evaluation

We use the Silhouette score [78] to evaluate the internal validity of subtypes obtained by a cancer subtyping method. The Silhouette score ranges from − 1 to 1 (the higher the Silhouette score, the better the method). The Silhouette score is the average of Silhouette widths over all samples. A Silhouette width is calculated based on the Euclidean distance or similarity and indicates how similar a sample is to its own subtype compared to other subtypes. A high value of Silhouette width indicates that the sample is well matched to its own subtype and poorly matched to other subtypes.

We use the concordance index (C-index) [79] to estimate the accuracy of a breast cancer prognosis method. C-index is defined as the proportion of all pairs of randomly chosen comparable patients in which the predictions and outcomes are concordant. For a pair of comparable patients, the concordance between predictions and outcomes means that the patient with the higher risk prediction experienced an event (e.g. death) before the one with the lower risk prediction. C-index ranges from 0 to 1. If the C-index of a method is equal to 0.5, that means this method is no better than a random guess method. C-index=1 means that the predictions and outcomes are perfectly concordant.

The Log-rank test [80] is used to assess the performance of both breast cancer subtyping and prognosis methods. The Log-rank test estimates whether the survival curves (estimated by the Kaplan–Meier (KM) survival method [81]) from two or more groups are identical or not. If the *p* value of the Log-rank test for a method on a given dataset is less than 0.05, we consider that the method can stratify patients in the dataset into several groups that have significantly different survival patterns. The groups correspond to subtypes in breast cancer subtyping methods or risk groups in breast cancer prognosis methods. The *p* value is an external validation measurement for breast cancer subtyping methods.

Besides these three evaluation metrics, we use two statistic tests to compare breast cancer prognosis methods. The Z-test [82] is used to assess whether a prognosis method significantly outperforms a random guess method. We also use the Cohen’s kappa coefficient to assess the concordance of the predictions between two breast cancer prognosis methods. Additional file 1 contains definitions of all the evaluation metrics and statistic tests.

### The CancerSubtypesPrognosis package

To streamline the evaluation, we develop a package (named CancerSubtypesPrognosis) that provides a pipeline of data pre-processing, feature selection, cancer subtyping, cancer prognosis, evaluation, and visualization. Data pre-processing includes distribution check, imputation, and normalization. Feature selection, including principal component analysis (PCA) [83], Variance, Cox model [84], and median absolute deviation (MAD), is used for removing irrelevant features. The CancerSubtypesPrognosis package offers 35 computational methods that are well-known in breast cancer subtyping or prognosis. There are 12 cancer subtyping methods and 23 computational methods for cancer prognosis in the package. To evaluate the results, CancerSubtypesPrognosis provides three statistical methods, the Silhouette score, Log-rank test, and C-index. Meta-analysis can be conducted by using Cohen’s kappa statistic in the package. The visualization tools in the package include the Kaplan–Meier (KM) survival curve, Silhouette plot, colour coded heat map [6], and the forest plot [85].

## Results

### Performance of the breast cancer subtyping methods based on multiple levels of transcriptomic data

We applied the cancer subtyping methods to different types of expression data, e.g. miRNA, lncRNA and mRNA, and combinations of them to explore whether miRNA/lncRNA data helps improve the performance of the methods. We used the *p* value derived from the Log-rank test and the Silhouette score to evaluate the performance of the subtyping methods.

#### Using miRNA expression data improves the performance of the breast cancer subtyping methods

Our experimental results show that the majority of methods perform better when using miRNA expression data alone or matched miRNA and mRNA (miRNA-mRNA) expression data in comparison with using mRNA expression data alone. For the sake of simplicity, we term “using miRNA expression data alone or miRNA-mRNA expression data” as “using miRNA expression data” in this paper. The experiments were conducted on the miRNA-mRNA expression data which are available in the TCGA753, METABRIC, UK, HEL and GSE19783 datasets (please refer to Table 1). We conclude that using miRNA expression data improves performance for breast cancer subtyping based on the following evidence.

**Fig. 1:**
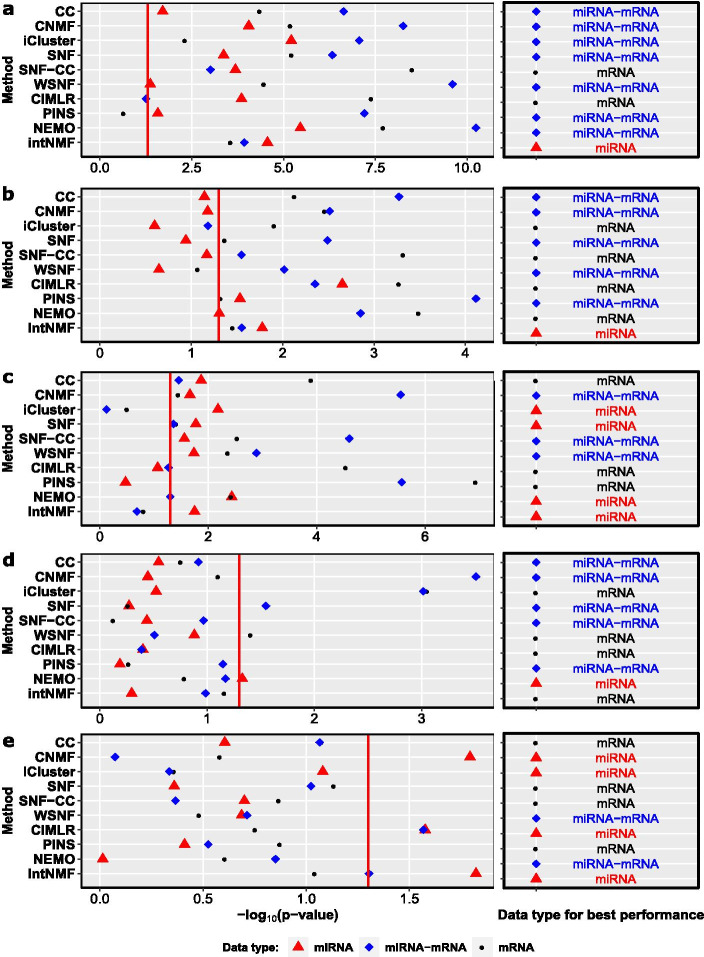
Performance of the methods when using mRNAs, miRNAs and both, respectively. **a** METABRIC (1283). **b** TCGA (753). **c** UK (207). **d** HEL (115). **e** GSE19783 (99). The x-axis of each diagram is the value of $-\log _{10}(\text {p-value})$, and the y-axis is the method. For better visibility, the scales of the x-axis of the diagrams are different. Black circles, red triangles, and blue diamonds denote the results of the methods when using mRNA data, miRNA data, and matched miRNA-mRNA data, respectively. Red vertical lines indicate the threshold for significantly different survival i.e. the results on the right of a red line are statistically significant, while the results on the left of the line are not

Firstly, using miRNA data can improve a method’s performance in detecting breast cancer subtypes with distinct survival patterns. We examine this by checking the *p* value of the Log-rank test of the cancer subtyping methods on each data type (i.e. mRNA data alone, miRNA data alone, and miRNA-mRNA data). Figure 1 shows the performance of ten methods using each of the five datasets. In each diagram in Fig. 1, the left panel shows the negative of log-transformed *p* value of each method on the three different types of data from the dataset. The right panel shows the data type with which the method achieves the best performance. PAM50 and IntClust are not applicable to miRNA data, so these two methods are not listed in Fig. 1. From the figure, it is clear that on the METABRIC, TCGA and HEL datasets, most methods achieve better performance when using miRNA-mRNA data than using mRNA data alone. For the UK dataset, four out of ten methods achieve significant results (*p* value < 0.05) using miRNA data alone. Meanwhile, only three out of ten mRNA-based methods achieve significant results. In three statistically significant results on GSE19783, two of them are obtained based on miRNA data alone, and one of them is from miRNA-mRNA data. Furthermore, we can see that in general the methods CC, CNMF, SNF, WSNF and PINS have benefited from miRNA-mRNA data, achieving more significant results than using mRNA data alone.

Secondly, most methods achieve higher Silhouette scores when using miRNA data alone on UK and HEL or using miRNA-mRNA data on METABRIC and TCGA than using mRNA data alone on the corresponding datasets (as shown in Additional file 1: Figure S1). When averaging the Silhouette scores of all the methods on mRNA data, miRNA data, and miRNA-mRNA data, respectively, the results show that using miRNA data alone has the largest average Silhouette score (0.579), followed by using miRNA-mRNA data (0.544) and using mRNA data alone (0.543). We see that CIMLR achieves the highest Silhouette scores on all the datasets and has been rarely affected by data types. The reason might be that CIMLR uses multiple Gaussian kernels to estimate similarity matrix instead of computing affinity matrix based on the Euclidean distances.

Thirdly, the cancer subtyping methods using miRNA data alone or miRNA-mRNA data perform better than the state-of-the-art mRNA-based methods PAM50 and IntClust in most cases. PAM50 and IntClust are two well-known breast cancer subtyping methods and therefore, it is important to know whether the other methods can achieve better results when using miRNA data alone or miRNA-mRNA data. Here, we focus on the clinical meaning of the results, so we compare the performance based on *p* values. From the results in Additional file 1: Figure S2, we can see that most methods outperform PAM50 on TCGA753, HEL, and GSE19783, and outperform IntClust on all the five datasets.

Additionally, the efficiency of the cancer subtyping methods can be improved by feature selection methods. There is a concern that including more data types could increase the time complexity of a method as it increases the dimensionality of the data. Therefore, in this study, we apply feature selection to the datasets to select the top 2000 mRNAs based on their median absolute deviation (MAD) values and the miRNAs/lncRNAs whose MAD values are bigger than 0.001. We have recorded the running time of all the cancer subtyping methods on the miRNA-mRNA data, as shown in Additional file 1: Table S1. We also show the computational time of PAM50 and IntClust on the mRNA data alone in this table. All the methods were run on a laptop with an i7-6600U CPU (2.8 GHz), and 16 GB of RAM. Most methods take (on average) less than 10 min on processing a dataset, except for CNMF, SNF-CC and intNMF (Additional file 1: Table S1). CNMF and intNMF are slower since they run (by default) the NMF method 150 times in order to compute consensus matrices. The SNF-CC method runs both SNF and CC methods, requiring extra time to compute the affinity matrix from a generic distance matrix.

#### Using lncRNA expression data alone achieves comparable performance with using mRNA expression data alone

**Fig. 2:**
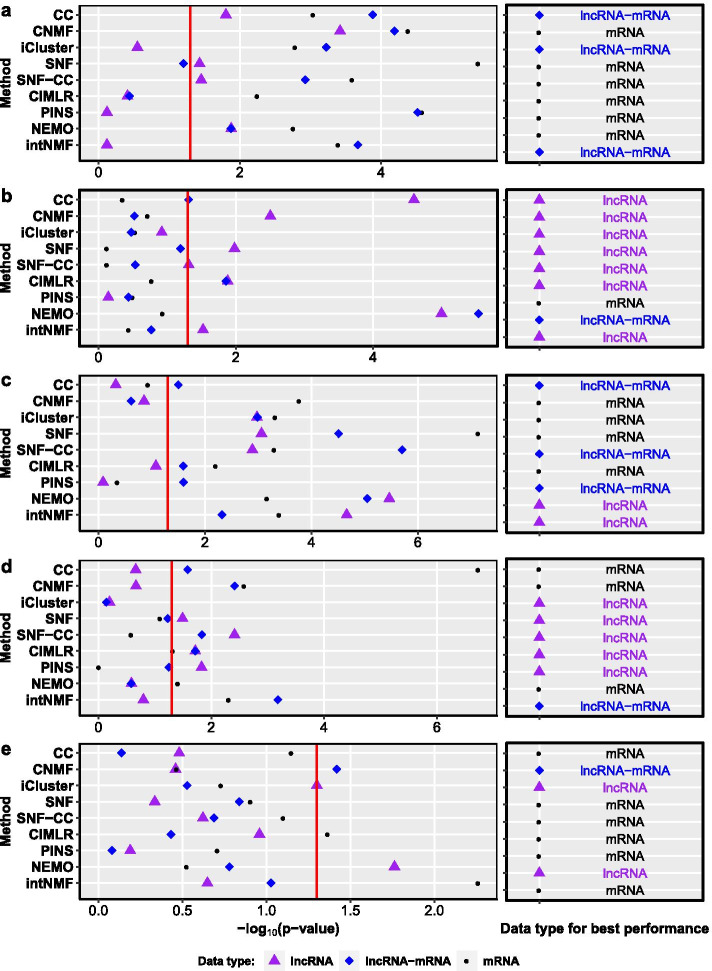

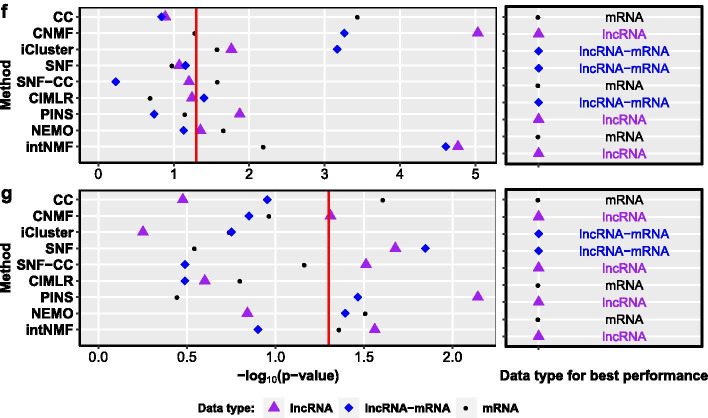
Performance of the methods when using mRNAs, lncRNAs and both, respectively. **a** GSE12276. **b** GSE19615. **c** GSE20685. **d** GSE20711. **e** GSE21653. **f** GSE42568. **g** GSE9195. The x-axis of each diagram is the value of $-\log _{10}(\text {p-value})$, and the y-axis is the method. Black circles, purple triangles, and blue diamonds denote the results of the methods on mRNA data, lncRNA data, and lncRNA-mRNA data, respectively

Similar to the above subsection, we compare the performance of the cancer subtyping methods based on lncRNA expression data alone, lncRNA-mRNA expression data and mRNA-data alone. GSE12276, GSE19615, GSE20685, GSE20711, GSE21653, GSE42568, and GSE9195 are first used for the comparison. We only consider the results which are statistically significant (the corresponding marks are on the right hand side of red lines in Fig. 2) here. We observe that using lncRNA expression data alone achieves the most significant results on GSE19615 (by 5 methods), GSE20711 (4) and GSE9195 (4). Using mRNA expression data alone achieves better results on GSE12276 (by 6 methods), GSE20685 (4) and GSE21653 (2) than using lncRNA data. In the remaining dataset (GSE42568), using the three data types can obtain similar performance. Besides, using lncRNA or lncRNA-mRNA expression data marginally outperforms gene-based methods PAM50 and IntClust according to the Log-rank tests (as shown in Additional file 1: Figure S4).

According to the internal validation measurement (Silhouette score), we observe that using lncRNA data alone can improve breast cancer subtyping methods on GSE20711 and GSE42568 (Additional file 1: Figure S3). Using lncRNA-mRNA data can lead to better results on GSE21653 than using mRNA data alone. On GSE12276, using three data types have similar performance. Unfortunately, using lncRNA data cannot improve the performance of most cancer subtyping methods on the remaining three datasets.

TCGA500 is the only dataset that contains matched lncRNA-miRNA-mRNA expression data. It is interesting to investigate the performance of cancer subtyping methods using miRNA and lncRNA expression data. Therefore, we compare the performance of the cancer subtyping methods based on individual omic data and different combinations of multi-omic data. From Additional file 1: Table S2, we observe that including miRNA data can improve breast cancer subtyping (*p* value: 0.0001) while including lncRNA data does not improve the performance of the cancer subtyping methods (*p* value: 0.045) on TCGA500. However, including lncRNA data achieve higher average Silhouette score (0.507) than using mRNA data alone (0.434) on TCGA500 (Additional file 1: Table S3).

### Performance of the breast cancer prognosis methods based on mRNA, miRNA, and lncRNA signatures

Different from the breast cancer subtyping methods, each breast cancer prognosis method is pre-trained on a fixed set of signatures, e.g. miRNAs. We use the trained model of each breast cancer prognosis method to predict the relative hazards (called risk scores) for patients in the applicable independent testing datasets. For instance, HOTAIR is applied to datasets with the lncRNA expression data of the HOTAIR signature. Additional file 1: Table S2 shows the applied datasets for each method. The running time for testing a breast cancer prognosis method on a dataset is shorter than 1 min. In this section, we use the C-index and the *p* value of the Log-rank test to measure the performance of these breast cancer prognosis methods on each dataset. By comparing the performance of the breast cancer prognosis methods, we can obtain the prognostic power of the different types of signatures, miRNAs, lncRNAs, and mRNAs. Finally, we assess the concordance between prognosis methods using the Cohen’s kappa statistic.

#### Multi-gene based prognosis methods perform better than single-gene based methods, miRNA-based methods, and lncRNA-based methods

**Fig. 3:**
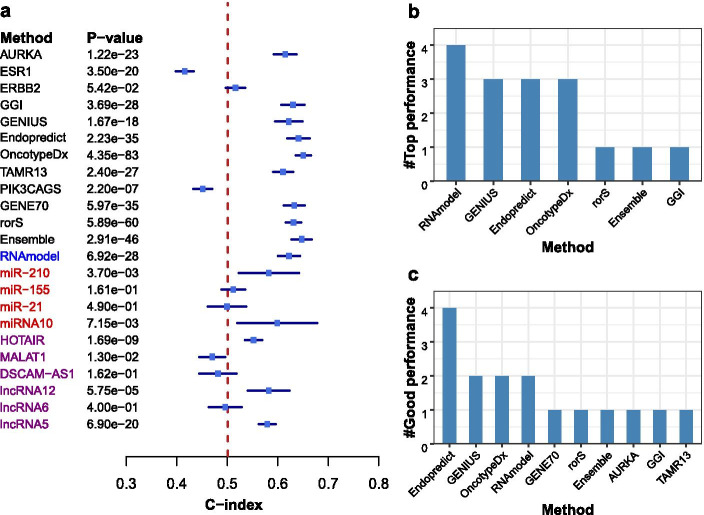
The meta-analysis of different breast cancer prognosis methods. **a** The forest plot of mean C-indices. The names and *p* values of methods are listed on the left, mean C-indices and the standard deviations are shown on the right. The names of gene-based methods, miRNA-based methods, miRNA-mRNA-based method, and lncRNA-based methods are colored in black, red, blue, and purple respectively. **b** The X-axis is methods and the Y-axis is the number of datasets on which a method achieved the highest C-index. **c** The X-axis is methods and the Y-axis is the number of datasets on which a method obtained good performance (C-index $> 0.7$)

We use the forest plot to visualize the mean and standard deviation of C-indices^6^ of each breast cancer prognosis method (Fig. 3a). Most multi-gene based methods have a C-index over 0.6, which is better than the mean C-indices of single-based methods ESR1 and ERBB2 (also shown in Additional file 1: Figure S5). The AURKA method achieves comparable performance (mean C-index 0.61) with the multi-gene based methods, which is consistent with the results in [20], but it is still inferior to most multi-gene based methods. The two miRNA-based methods, miR-210 and miRNA10 get mean C-indices of 0.58 and 0.60 respectively, but they have higher standard deviations compared to other methods. Most multi-gene based methods perform better than most lncRNA-based methods (Their mean C-indices are less than 0.6). Furthermore, the OncotypeDX method has the highest mean C-index (0.65) and lowest standard deviation (0.008), which implies that OncotypeDX can produce more accurate and robust results than other methods in our experiments.

We use the Z-test to test whether a method is significantly better or worse than a random guess. The null hypothesis is that there is no difference between the performance (mean C-index) of the method and the performance of the random guess (whose mean C-index is 0.5). The one-sided *p* values of methods are listed in Fig. 3a. The multi-gene based methods are significantly better than the random guess and they are also superior to miRNA-based methods and lncRNA-based methods. However, the *p* values of the ERBB2, miR-155, miR-21, and lncRNA6 methods are higher than 0.05 which indicates that these methods do not outperform the random guess. ESR1, PIK3CAGS and MALAT1 are inferior to the random guess on the testing datasets.

As mean C-index can be biased by outliers in the C-indices, we also investigate the C-index of a method on each dataset. The results are shown in Additional file 1: Figure S5. We count the number of datasets on which a method has the highest C-index among all the methods and the number of datasets on which a method obtains good performance (C-index > 0.7). The results are shown in Fig. 3b, c respectively. To provide a fair comparison, we exclude the results of the methods on their training datasets (including selecting signatures and estimating the coefficients in the models). For example, miRNA10 and RNAmodel were trained on UK and a subset of TCGA data, respectively. For this reason, these methods may have good performances on UK, TCGA753, or TCGA500. The top methods which obtained the highest C-index are RNAmodel (on 4 datasets), GENIUS (3), EndoPredict (3), OncotypeDX (3), rorS (1), Ensemble (1) and GGI (1). The top three performers with C-index > 0.7 are EndoPredict (on 4 datasets), GENIUS (2), OncotypeDX (2), RNAmodel (2), GENE70 (1), rorS (1), Ensemble (1), AURKA (1), GGI (1) and TAMR13 (1). The results based on individual C-index are consistent with the conclusion drawn based on the mean C-indices.

#### Most methods can successfully stratify patients into two risk groups with significantly different survival patterns, but the concordance between the methods on individual patient risk group prediction is low

**Fig. 4:**
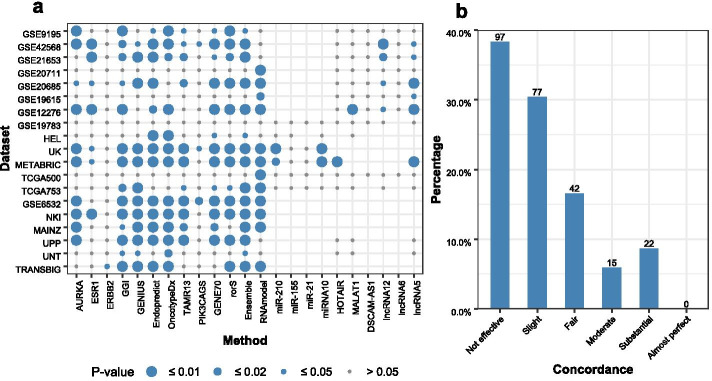
Results of the Log-rank test and the Cohen’s kappa statistic. **a** The *p* values by the Log-rank test. The x-axis is methods and the y-axis is datasets. A steel blue point means that the *p* value of the method is less than 0.05, which indicates that the method can successfully stratify patients in the dataset into two risk groups with significantly different survival patterns. A (small) grey point means the two risk groups are not significantly different between each other by the method on the dataset. **b** The histogram of concordance values estimated using the Cohen’s kappa statistic. There are six levels of concordance values between methods (More information can be found at Additional file 1)

Based on the predicted risk scores from a breast cancer prognosis method, patients can be stratified into a high-risk group or low-risk group. If a patient’s risk score is bigger than the median risk score of the cohort, the patient is put into the high-risk group, otherwise, the patient is put into the low-risk group. We use the Log-rank test to assess the difference in survival pattern between the high-risk group and the low-risk group. Figure 4a shows that Ensemble (on 15 datasets), GGI (14), OncotypeDX (13), EndoPredict (13), GENE70 (13), GENIUS (12), rorS (12), RNAmodel(12), TAMR13 (11), and AURKA(10) can successfully stratify the patients in most datasets into two risk groups with significantly different survival patterns. On the contrary, miR-155, miR-21, DSCAM-AS1, and lncRNA6 cannot stratify patients in the 19 datasets into two risk groups with distinct survival patterns. It is important to note that we apply the mRNA signatures in RNAmodel on the datasets that don’t contain miRNA expression data. The good performance of RNAmodel in our results indicates the mRNA signatures in RNAmodel are effective for predicting breast cancer risk groups. There is not enough evidence that miRNA/mRNA mixture signatures in RNAmodel outperform other multi-gene signatures based on the results on the six datasets with miRNA-mRNA expression data (Fig. 4a).

It is expected that these prognosis methods would have a good percentage of agreement with each other on the prediction of an individual patient’s risk group for determining the treatment strategy to be applied to the patient. To verify this, we estimate the concordance (i.e. the percentage of agreement) between two prognosis methods for risk group prediction for individual patients. The concordance between a pair of methods is estimated by using the Cohen’s kappa statistic, and the results are shown in Fig. 4b and Additional file 1: Figure S7. Based on the result of the Cohen’s kappa test, we divide the degree of agreement between methods into six levels. Surprisingly, 38.3% of the method pairs are tested to have no effective agreement (kappa value less than 0) with each other on individual patients’ risk group predictions, and 30.4% of the method pairs slightly agree (kappa value is between 0 and 0.2) with each other. There are 16.6% of the method pairs which fairly agree (kappa value is between 0.2 and 0.4) with each other, 5.9% or 15 method pairs moderately agree (kappa value is between 0.4 and 0.6) with each other, 8.6% or 22 method pairs substantially agree (kappa value is between 0.6 and 0.8) with each other. None method pairs almost perfectly agree (kappa value is between 0.8 and 1) with each other. The results show that most of the analysed computational methods for breast cancer prognosis do not have a good percentage of agreement for the risk group prediction. As a result, it can be difficult for clinicians to define a personalised treatment schedule based on the patient’s risk group prediction made by these methods.

#### The roles of miRNA/lncRNA signatures in the improvement of breast cancer prognosis needs to be further verified

We further evaluate the average performance of all the methods on three different data types (mRNA data, miRNA data, and lncRNA data), respectively. The forest plots of the mean C-indices of the methods on the three data types are shown in Additional file 1: Figure S6. Figure S6 shows that using miRNA or lncRNA data alone results in worse performance in comparison to use mRNA data alone. We can safely infer that breast cancer prognosis methods based on miRNA/lncRNA data do not outperform methods using gene expression data.

The prognostic roles of some miRNA/lncRNA signatures in breast cancer are not clear. Although studies have shown that miR-21 and miR-155 function as oncogenes in breast cancer [72, 86], their expression level is not significantly negatively correlated with survival outcomes based on our results. Similarly, MALAT1 and DSCAM–AS1 are found to over-expressed in breast cancer patients with poor outcomes [76, 87], unfortunately, we cannot observe this information from our results.

However, some miRNA and lncRNA signatures do have prognostic value for breast cancer. For example, the C-indices of miRNA10 are higher than 0.5 in its independent testing datasets (Additional file 1: Figure S5), which means the predicted hazard risk scores are consistently negatively correlated with survival outcomes. lncRNA12 and lncRNA5 perform well on the datasets produced by Affymetrix Human Genome U133 Plus 2.0 arrays. From the results (Additional file 1: Figure S5), it can be seen that the up-regulated expression of miR–210 is related to poor prognosis (c-index is higher than 0.5) within most breast cancer cohorts, which has evidenced by a quantitative real-time PCR (qRT-PCR) experiment recently [88]. Similarly, the expression level of HOTAIR is positively correlated with hazard risk, which implies HOTAIR could be an oncogenic lncRNA in breast cancer [89].

## Discussion

We have evaluated the usefulness of non-coding RNAs (miRNAs and lncRNAs) expression data for improving the performance of breast cancer subtyping and prognosis by benchmarking the state-of-the-art methods on multiple levels of transcriptomic data. In contrast to previous comparisons [25, 26, 61, 90], we focused on the evaluation of the breast cancer subtyping and prognosis results based on different data types. Existing comparisons for cancer subtyping methods mainly used datasets from different cancer types and aimed to find out the best method(s) [25, 61, 90], however, in our work, we have tried to explore the best expression data types for the methods. Existing comparison for cancer prognosis methods was limited to gene-based methods [26], while we here further considered different independent testing datasets, as well as miRNA-based and lncRNAs-based methods.

The experimental results showed that the cancer subtyping methods using miRNA-mRNA data outperformed the mRNA-based methods, including PAM50 and IntClust, for breast cancer subtyping. However, the cancer subtyping methods using lncRNA data did not display better performance than these methods using mRNA data alone. Hence, mRNA/miRNA expression data rather than lncRNA expression data should be prioritized by researchers on the present RNA expression microarray datasets.

Interestingly, we also observed that current cancer subtyping methods cannot guarantee that the identified subtypes have distinct survival patterns. For example, most methods showed good Silhouette scores but were not be able to group patients into subtypes with distinct survival patterns (as shown in Fig. 1d–e and Additional file 1: Figure S1d–e). This can be due to the fact that most computational methods are designed to cluster the samples with similar features (instead of similar survival pattern) into the same group. In other words, from the computational point of view, a positive Silhouette score indicates that a method is effective to cluster samples into groups with distinct feature patterns. However, biomedical researchers more focus on whether the cancer subtypes have prognostic significance. Therefore, a novel method is required to take the prognostic significance of subtypes into account when training the model in the future.

When the clinical data is available, we suggest using the external validity (*p* value) of clusters to evaluate the prognostic performance of a cancer subtype method. To discover distinct breast cancer subtypes from miRNA-mRNA expression data, the recommended methods are SNF and CNMF, whose results had the best external validity in our evaluation. As shown in Fig. 1, SNF and CNMF successfully stratified patients in METABRIC, TCGA753, UK and HEL into subtypes with distinct survival patterns, while other methods only succeeded in three datasets or less.

Since many of the current data does not include long term follow-up clinical data for patients, another metric to evaluate the cluster results is required. To this end, we can use the Silhouette score to assess the internal validity of clustering results. Based on the internal validity, the preferred method is CIMLR, which achieved the highest consistency within clusters of gene expression data. Please let us highlight this paper aims to uncover the roles of miRNAs/lncRNAs in characterising breast cancer subtypes and prognosis. Complementary information about the theory and other benchmarking of multi-omic cancer subtyping methods can be found in [25, 61, 90].

In addition to the evaluation metrics discussed here, downstream analysis can also assist method and data type selection via interpreting the biological meaning of the cancer subtyping results. For example, differential gene expression analysis can be used to obtain differentially expressed genes and miRNAs in different clusters. Thus, we have included differential gene expression analysis methods in the CancerSubtypesPrognosis package.

For breast cancer prognosis, we suggest using multi-gene based methods including EndoPredict, RNAmodel, OncotypeDX, GENIUS, GGI, and rorS for breast cancer prognosis, since these six methods appeared in both the top performer and the good performer list. If all the signatures in the five methods (GENIUS, EndoPredict, OncotypeDX, GENE70, and rorS) are available, we also suggest using the Ensemble method in our package as the ensemble method outperformed all the other methods for stratifying patients into two distinct risk groups. We noted that ESR1, ERBB2, PIK3CAGS might not work on the whole breast cancer cohort even though they have been found to be effective in specific breast cancer cohorts. It is interesting to investigate why some methods work well on some datasets while not well on others. Although we do not have evidence to support our arguments, we think that the genes in the signatures may not be the causal genes, i.e. genes causing the disease, and therefore the performance of methods using those signatures are not robust across different datasets [91].

We also observed that current breast cancer prognosis methods (even multi-gene based methods) do not have high concordance on patient risk group prediction. The reason might be current methods are not good enough for breast cancer prognosis yet. Improving the performance of methods for breast cancer prognosis will ultimately improve the concordance between them.

In this regard, we suggest considering the following two points of view to improve the performance of breast cancer prognosis methods. From the computational perspective, advanced machine learning and artificial intelligence algorithms can be used to catch non-linear relationships between gene expression profiles and survival outcomes [92]. From the biological perspective, combining gene, miRNA and lncRNA signatures has the potential to improve breast cancer prognosis and help researchers understand the biological mechanisms involved. This is based on the fact that the heterogeneity of breast cancer is caused by diverse molecular mechanisms, including gene mutation [93], miRNA regulation [94], lncRNA regulation [95] or competing endogenous RNAs (lncRNAs-associated) [96]. Even though some miRNAs/lncRNAs were not significantly associated with survival outcomes, the prognostic value of some miRNAs/lncRNAs was evidenced in our study and previous studies.

Unfortunately, it is difficult and costly to obtain multiple omics data for the same patients. Affymetrix HG-U133 Plus 2.0 arrays included a small part but not all of the possible lncRNAs in the human genome. Limited datasets and incomplete lncRNA expression data may not allow us to comprehensively elucidate the roles of lncRNAs in breast cancer prognosis. However, RNA sequencing technology is becoming mature and cheap, which will be allowed to significantly increase omics data access in the future. We will be able to evaluate a more comprehensive number of miRNA/lncRNA signatures for breast cancer prognosis based on these data.

## Conclusion

We have evaluated the usefulness of using miRNA/lncRNA data for breast cancer subtyping and prognosis by conducting a comprehensive comparison of 35 computational methods on 19 breast cancer datasets. The comparison study showed that using miRNA data improves the performance of the current cancer subtyping methods, while using lncRNA data have similar performance to using mRNA data alone. Current miRNA/lncRNA signatures do not outperform multi-gene based breast cancer prognosis methods. We acknowledge that our conclusions are limited by the number of methods and datasets used in the evaluation, but we believe that the results can provide valuable clues about the roles of miRNA/lncRNA in characterising breast cancer subtypes and prognosis.

For the convenience of researchers to apply these methods to new datasets, we have released an R package named CancerSubtypesPrognosis. CancerSubtypesPrognosis is flexible and can easily be extended to different data types such as genomic, transcriptomic and epigenomic data. We hope the package can help the application and evaluation of existing methods and the development of new breast cancer subtyping and prognosis methods.

## Supplementary Information

**Additional file 1**. Supplementary tables, figures and note.

## Data Availability

The CancerSubtypesPrognosis package, the R source codes, and the datasets to reproduce results in this work are freely available in the GitHub, (https://github.com/XiaomeiLi1/CancerSubtypesPrognosis).

## Abbreviations

BC: Breast cancer
ER+: Estrogen receptor-positive breast cancer
C-index: Concordance index

## Footnotes

1 http://firebrowse.org/.

2 https://www.ebi.ac.uk/ega/.

3 https://bioconductor.org/.

4 https://www.ncbi.nlm.nih.gov/geo/.

5 https://www.gencodegenes.org/.

6 The mean C-index is the average C-index of a method on its applicable datasets.
