# Supplementary material for: Uncovering the roles of microRNAs/lncRNAs in characterising breast cancer subtypes and prognosis
Source: BMC Bioinformatics. 2021 Jun 4;22:300. doi: 10.1186/s12859-021-04215-3 (PMC8176586; doi:10.1186/s12859-021-04215-3)
Supplement: Supplementary file 1 — Additional file 1. Supplementary tables, figures and note. [file 12859_2021_4215_MOESM1_ESM.pdf]

Supplement to "Uncovering the roles of microRNAs/lncRNAs in characterising breast cancer subtypes and prognosis"

Xiaomei Li[1], Buu Truong[1], Taosheng Xu[2], Lin Liu[1], Jiuyong Li[1] and Thuc Duy Le[1,3,*]

1 UniSA STEM, University of South Australia, Mawson Lakes, SA 5095, Australia
2 School of Life Sciences, University of Science and Technology, Hefei, Anhui 230021, China,
3 Centre for Cancer Biology, University of South Australia, Adelaide, SA 5000, Australia,
* Thuc.Le@unisa.edu.au

Table S1: **The runtime of each cancer subtyping method on multi-omic data (seconds).**

	PAM50	IntClust	CC	CNMF	iCluster	SNF	SNF-CC	WSNF	CIMLR	PINS	NEMO	intNMF
TCGA753	0.4	6.7	44.2	3458.7	216.4	30.6	1256.2	330.3	325.6	174.6	9.9	1530.0
METABRIC	0.9	16.6	124.9	4110.0	334.7	140.0	6251.2	1101.2	1388.3	411.6	37.6	3915.7
UK	0.3	9.9	4.6	495.7	213.1	1.5	68.5	181.7	63.8	16.9	1.3	480.8
HEL	0.2	10.0	1.8	541.1	239.2	0.6	31.7	103.8	48.5	17.8	0.5	259.6
GSE19783	0.1	6.9	2.2	458.4	326.9	0.4	27.1	55.5	38.4	20.1	0.3	180.4
TCGA500	0.3	7.6	30.7	2059.6	1173.2	13.6	388.9	213.5	175.3	81.4	7.5	1222.3
GSE12276	0.4	7.6	5.3	724.6	1013.0	2.1	63.9	114.3	34.6	13.0	1.7	473.7
GSE19615	0.3	7.7	2.2	375.3	1005.4	0.6	28.1	66.6	21.8	5.6	0.5	166.5
GSE20685	0.2	8.6	11.8	1223.4	795.5	4.4	156.7	179.5	71.7	87.3	2.8	777.7
GSE20711	0.4	7.6	2.0	299.0	809.2	0.4	20.0	49.1	34.5	5.7	0.3	160.4
GSE21653	0.2	7.7	7.5	924.6	814.2	2.4	92.9	144.7	45.3	53.6	2.2	595.5
GSE42568	0.1	8.0	2.0	218.9	228.8	0.5	25.0	58.8	21.9	8.4	0.4	200.3
GSE9195	0.2	9.5	1.9	289.5	584.1	0.3	15.8	48.2	28.8	5.2	0.3	131.5

Note: the computational time of PAM50 and IntClust was based on the mRNA data of the corresponding dataset, the same to WSNF on TCGA500, GSE12276, GSE19615, GSE20685, GSE20711, GSE21653, GSE42568 and GSE9195.

Table S2: **The p-values of cancer subtyping methods based on the TCGA500 dataset.**

Method	mRNA	miRNA	lncRNA	mRNA/miRNA	mRNA/lncRNA	miRNA/lncRNA	mRNA/miRNA/lncRNA
CC	0.001	0.101	**0.054**	0.379	0.085	0.606	**0.035**
CNMF	0.166	0.589	0.969	0.144	0.369	0.841	0.346
iCluster	0.210	0.192	0.260	0.017	**0.045**	**0.047**	0.128
IntClust	0.710	NA	NA	NA	NA	NA	NA
SNF	0.995	0.240	0.384	0.482	0.971	0.751	0.751
SNF-CC	0.099	0.285	0.756	0.284	0.552	0.358	0.236
WSNF	0.866	0.255	NA	0.390	NA	NA	NA
CIMLR	0.181	**0.089**	0.397	0.231	0.770	0.072	0.235
PINS	0.953	0.521	0.285	0.033	0.520	0.285	0.250
NEMO	0.079	0.121	0.482	0.427	0.748	0.348	0.479
intNMF	**0.0002**	0.874	0.203	**0.0001**	0.502	0.131	0.383
PAM50	0.946	NA	NA	NA	NA	NA	NA

NA: an inability of the corresponding method to apply on the data. The best result for each data is highlight with **bold**.

Table S3: **The Silhouette scores of cancer subtyping methods based on the TCGA500 dataset.**

Method	mRNA	miRNA	lncRNA	mRNA/miRNA	mRNA/lncRNA	miRNA/lncRNA	mRNA/miRNA/lncRNA
CC	0.55	0.58	0.57	0.49	0.51	0.54	0.49
CNMF	0.85	0.27	0.68	0.78	0.67	0.64	0.66
iCluster	0.31	0.27	0.06	0.32	0.32	0.30	0.32
IntClust	0.27	NA	NA	NA	NA	NA	NA
SNF	0.30	0.38	0.45	0.56	0.56	0.52	0.51
SNF-CC	0.63	0.89	0.81	0.86	0.85	0.87	0.89
WSNF	0.33	0.35	NA	0.56	NA	NA	NA
CIMLR	**1.00**	**1.00**	**1.00**	**1.00**	**0.99**	**0.99**	**1.00**
PINS	0.48	0.42	0.75	-0.11	0.31	0.75	0.31
NEMO	0.63	0.61	0.63	0.54	0.54	0.50	0.51
intNMF	0.76	0.86	0.94	0.83	0.87	0.86	0.90
PAM50	0.50	NA	NA	NA	NA	NA	NA
Average	0.434	0.327	0.421	0.239	**0.507**	0.382	0.340

NA: an inability of the corresponding method to apply on the data. The best result for each data is highlight with **bold**.

Table S4: **The applied datasets for each method.**

Method	TCGA753	METABRIC	UK	HEL	GSE19783	TCGA500	TRANSBIG	UNT	UPP	MAINZ	NKI	GSE6532	GSE12276	GSE19615	GSE20685	GSE20711	GSE21653	GSE42568	GSE9195
rorS	✓	✓	✓	✓	✓	✓	✓	✓	✓	✓	✓	✓	✓	✓	✓	✓	✓	✓	✓
GENE70	✓	✓	✓	✓	✓	✓	✓	✓	✓	✓	✓	✓	✓	✓	✓	✓	✓	✓	✓
OncotypeDX	✓	✓	✓	✓	✓	✓	✓	✓	✓	✓	✓	✓	✓	✓	✓	✓	✓	✓	✓
GGI	✓	✓	✓	✓	✓	✓	✓	✓	✓	✓	✓	✓	✓	✓	✓	✓	✓	✓	✓
Tamr13	✓	✓	✓	✓	✓	✓	✓	✓	✓	✓	✓	✓	✓	✓	✓	✓	✓	✓	✓
AURKA	✓	✓	✓	✓	✓	✓	✓	✓	✓	✓	✓	✓	✓	✓	✓	✓	✓	✓	✓
ESR1	✓	✓	✓	✓	✓	✓	✓	✓	✓	✓	✓	✓	✓	✓	✓	✓	✓	✓	✓
ERBB2	✓	✓	✓	✓	✓	✓	✓	✓	✓	✓	✓	✓	✓	✓	✓	✓	✓	✓	✓
GENIUS	✓	✓	✓	✓	✓	✓	✓	✓	✓	✓	✓	✓	✓	✓	✓	✓	✓	✓	✓
PIK3CAGS	✓	✓	✓	✓	✓	✓	✓	✓	✓	✓	✓	✓	✓	✓	✓	✓	✓	✓	✓
EndoPredict	✓	✓	✓	✓	✓	✓	✓	✓	✓	✓	✓	✓	✓	✓	✓	✓	✓	✓	✓
Ensemble	✓	✓	✓	✓	✓	✓	✓	✓	✓	✓	✓	✓	✓	✓	✓	✓	✓	✓	✓
RNAmodel	✓	✓	✓	✓	✓	✓	✓	✓	✓	✓	✓	✓	✓	✓	✓	✓	✓	✓	✓
miR-210	✓	✓	✓	✓	✓	✓													
miR-155	✓	✓	✓	✓	✓	✓													
miR-21	✓	✓	✓		✓	✓													
miRNA10	✓	✓	✓	✓	✓	✓													
HOTAIR	✓	✓			✓	✓		✓	✓				✓	✓	✓	✓	✓	✓	✓
MALAT1	✓			✓	✓	✓		✓	✓				✓	✓	✓	✓	✓	✓	✓
DSCAM-AS1					✓	✓							✓	✓	✓	✓	✓	✓	✓
lncRNA12	✓				✓	✓							✓	✓	✓	✓	✓	✓	✓
LncRNA6						✓							✓	✓	✓	✓	✓	✓	✓
LncRNA5	✓	✓			✓	✓		✓	✓				✓	✓	✓	✓	✓	✓	✓

✓: an ability of the corresponding method to apply on the dataset.

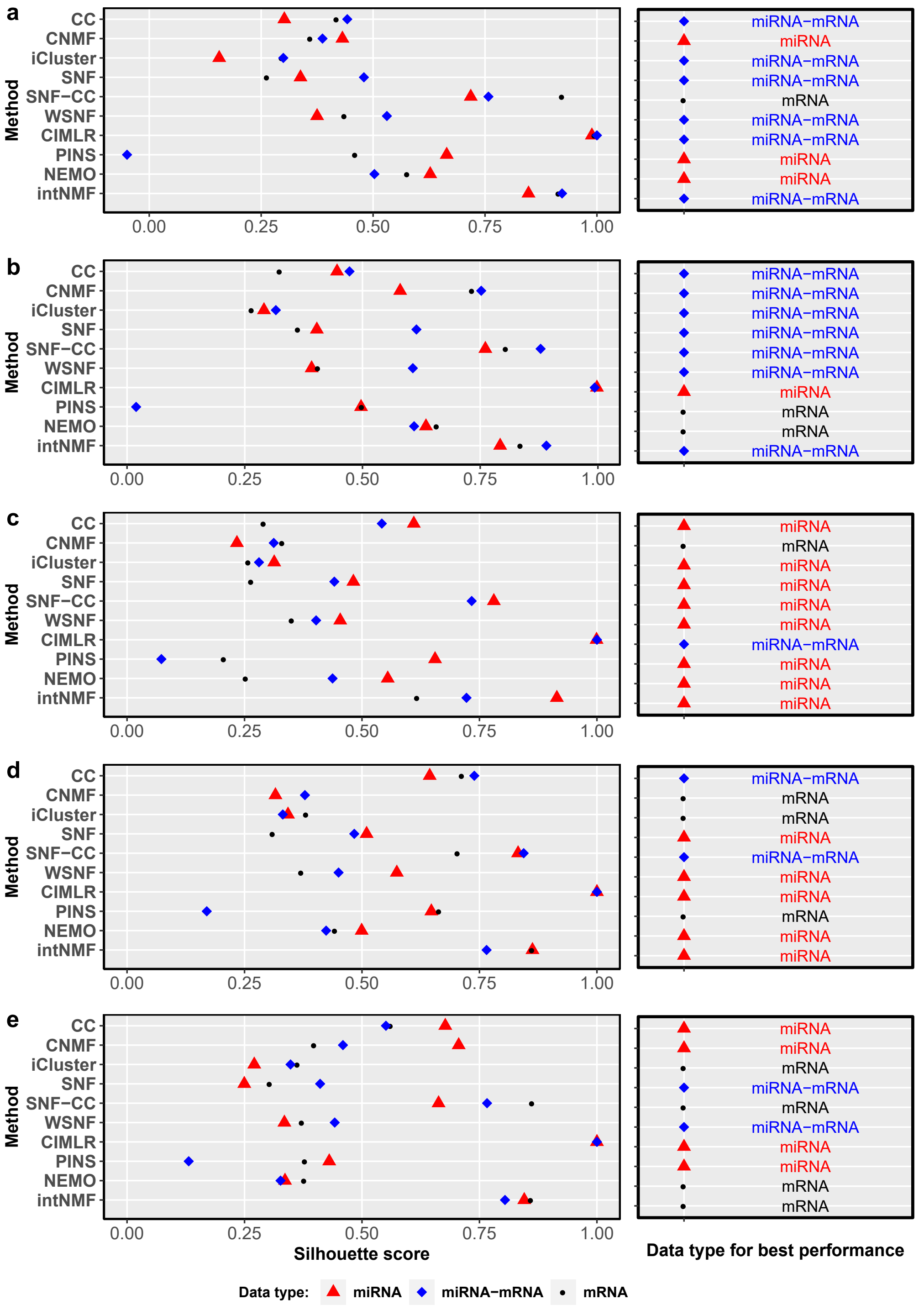

Figure S1: **The Silhouette scores of methods when using mRNAs, miRNAs and both, respectively. a.** METABRIC (1283). **b.** TCGA (753). **c.** UK (207). **d.** HEL (115). **e.** GSE19783 (99). The x-axis of each diagram is the Silhouette score, and the y-axis is methods. Red triangle, blue diamond, and black circle denote the results of a method on the miRNA data, the matched miRNA-mRNA data and mRNA data, respectively.

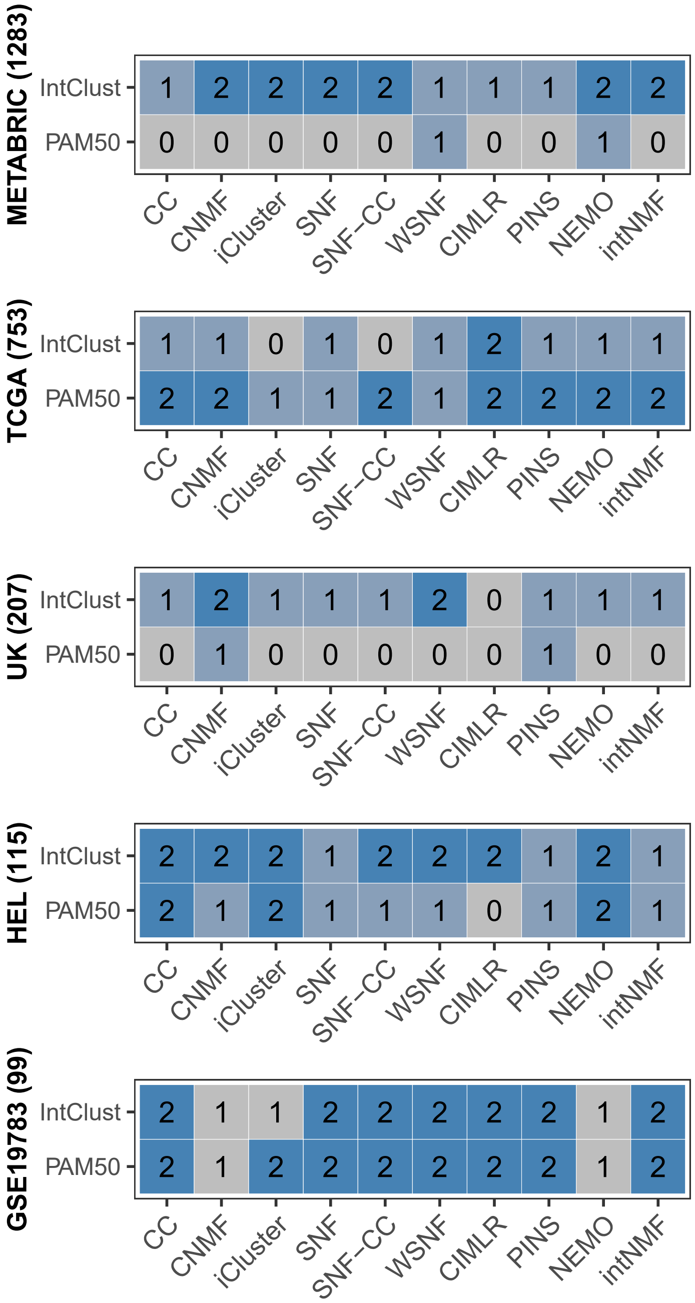

Figure S2: **The comparison of current multi-omic methods and the benchmark methods (p-value).** The number in the tile indicates how many data types (miRNAs-mRNAs and/or miRNAs) used by a multi-omic method outperforms the benchmark method (PAM50 or IntClust).

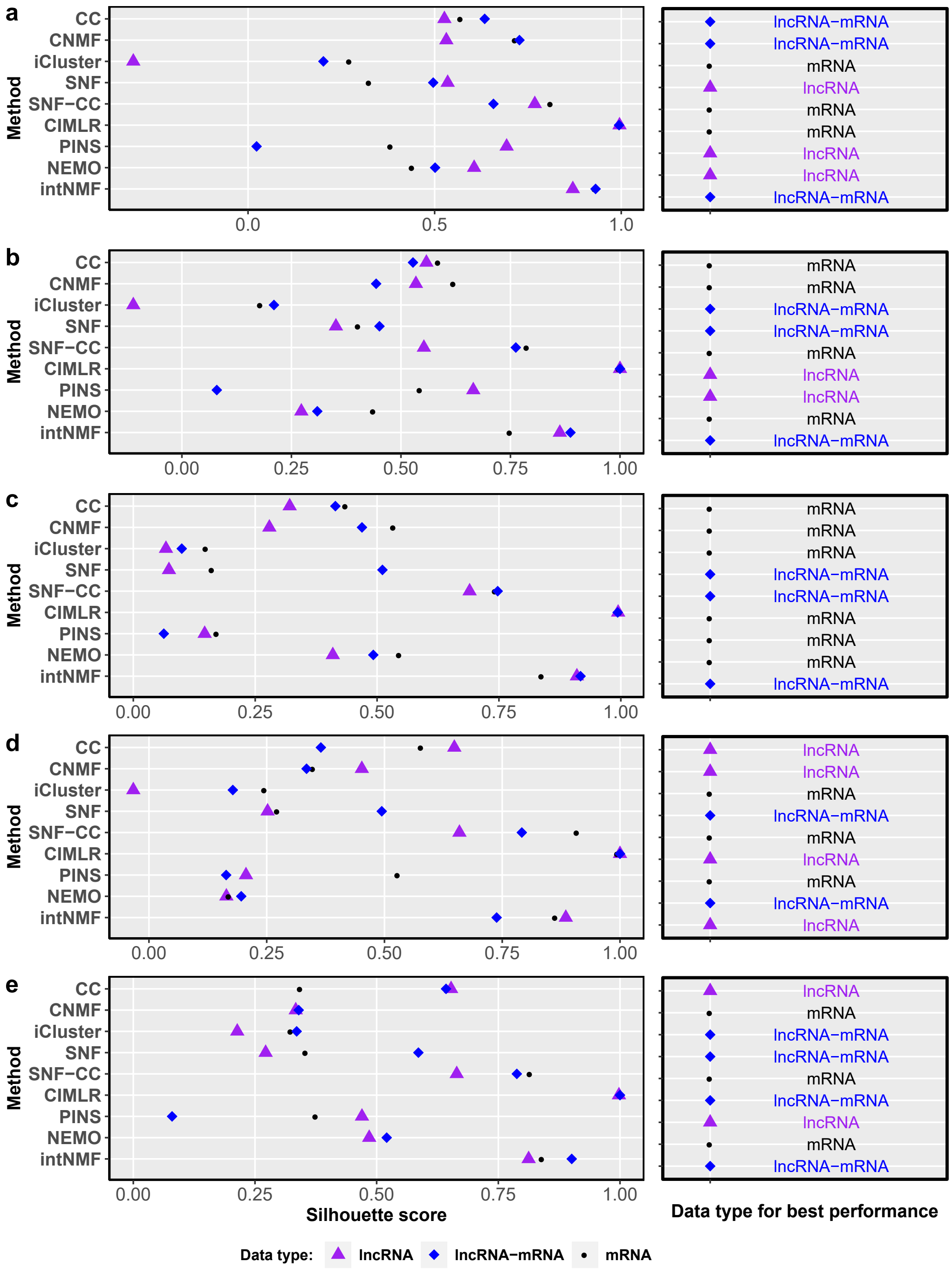

Figure S3: **The Silhouette scores of methods when using mRNAs, lncRNAs and both, respectively. a.** GSE12276. **b.** GSE19615. **c.** GSE20685. **d.** GSE20711. **e.** GSE21653. The x-axis of each diagram is the Silhouette score, and the y-axis is methods. Purple triangle, blue diamond, and black circle denote the results of a method on the lncRNA data, the matched lncRNA-mRNA data and mRNA data, respectively.

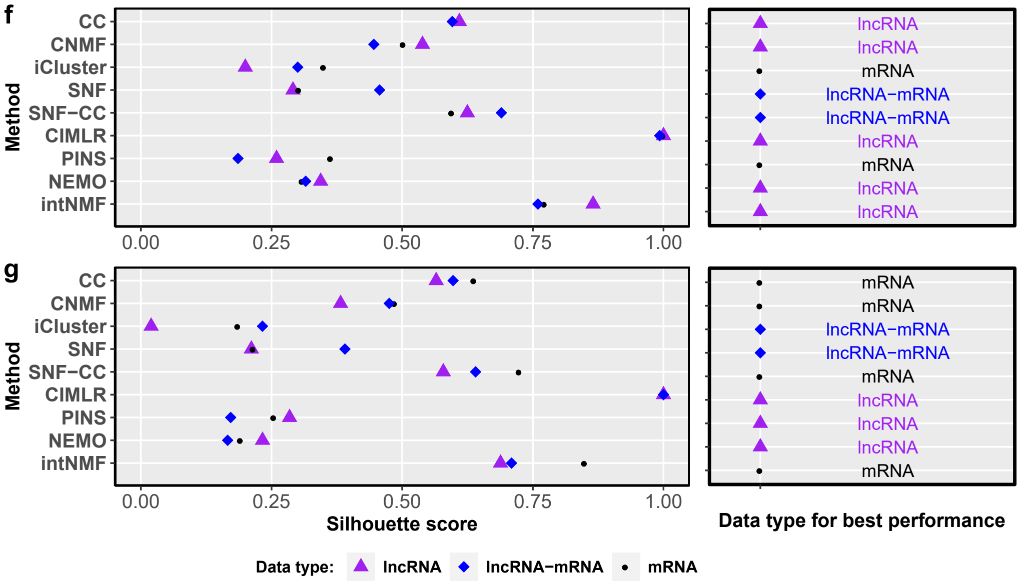

Figure S3: **The Silhouette scores of methods when using mRNAs, lncRNAs and both, respectively (continued). f.** GSE42568. **g.** GSE9195. The x-axis of each diagram is the Silhouette score, and the y-axis is methods. Purple triangle, blue diamond, and black circle denote the results of a method on the lncRNA data, the matched lncRNA-mRNA data and mRNA data, respectively.

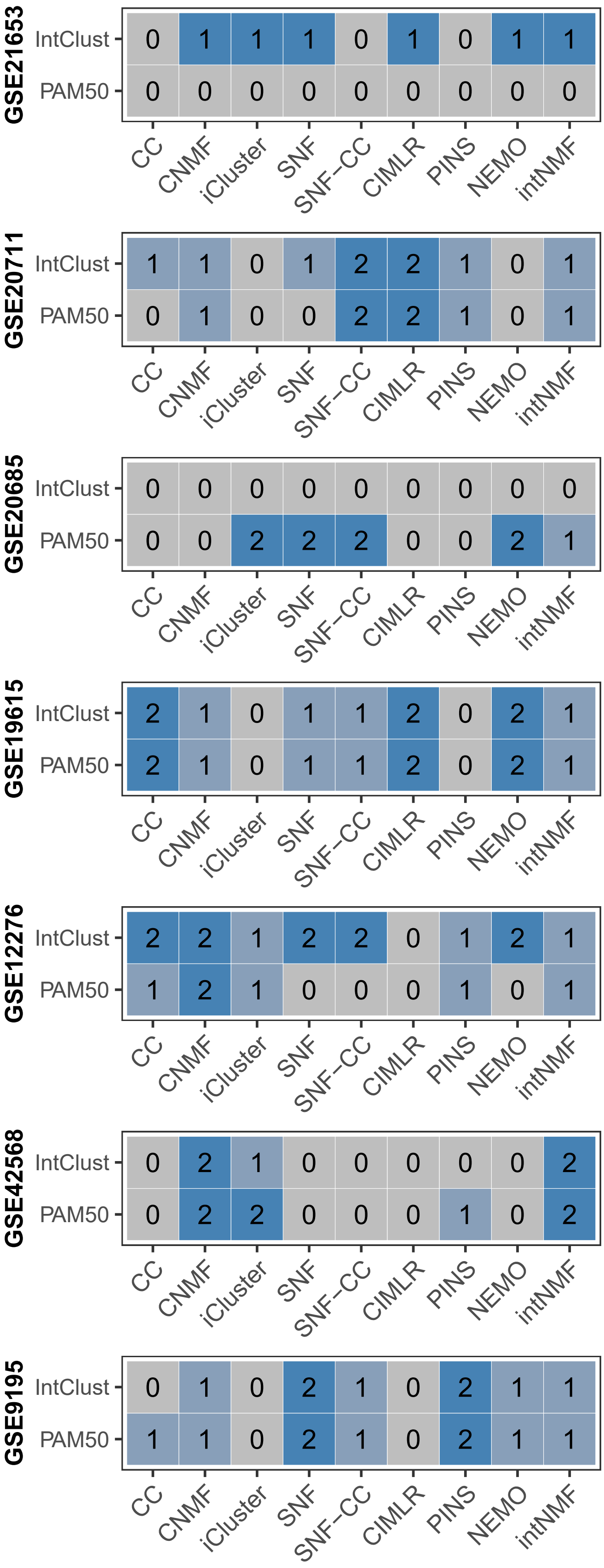

Figure S4: **The comparison of current multi-omic methods and the benchmark methods (p-value).** The number in the tile indicates how many data types (lncRNAs-mRNAs and/or lncRNAs) used by a multi-omic method outperforms the benchmark method (PAM50 or IntClust).

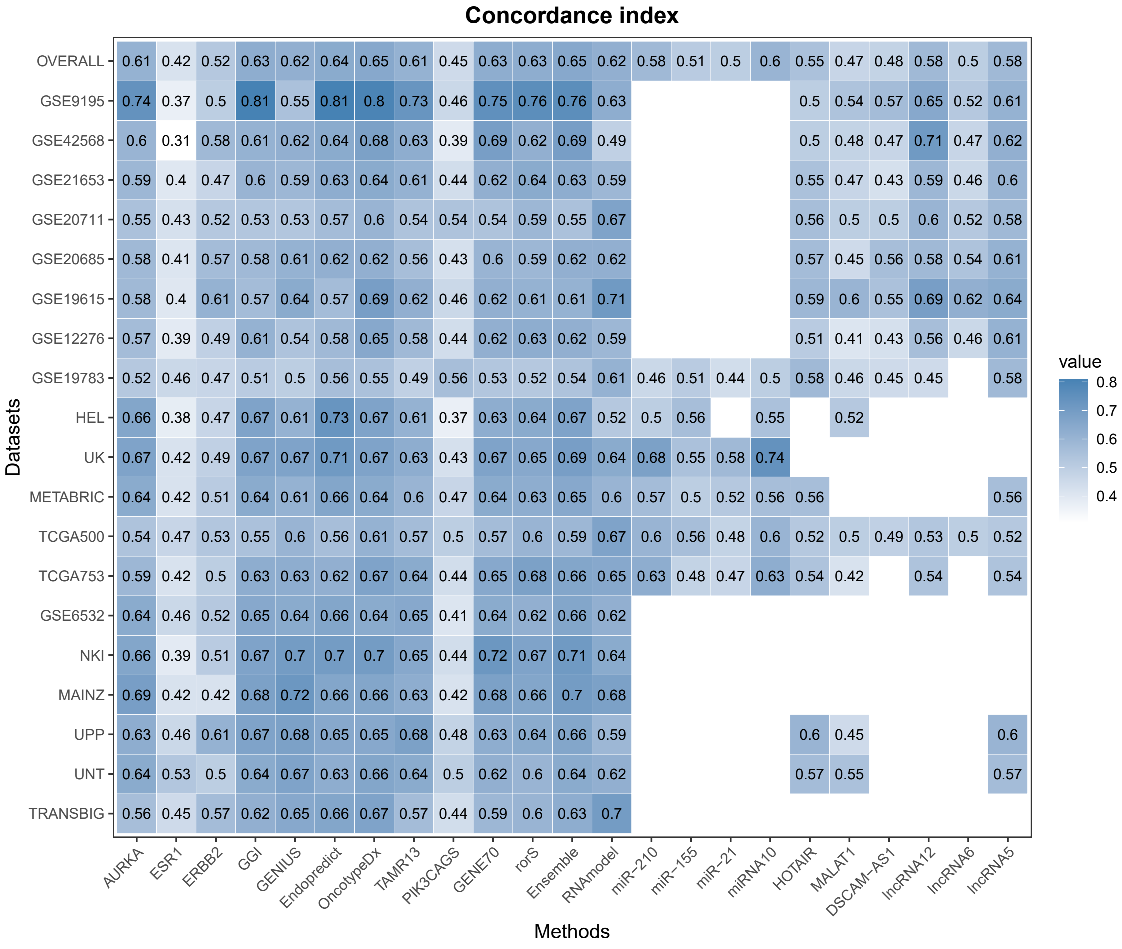

Figure S5: **Performance of all the cancer prognosis methods on 13 breast cancer datasets (C-indices).** The C-indices are colored from white to steel blue. An empty field means an inability of the corresponding method to predict on the dataset.

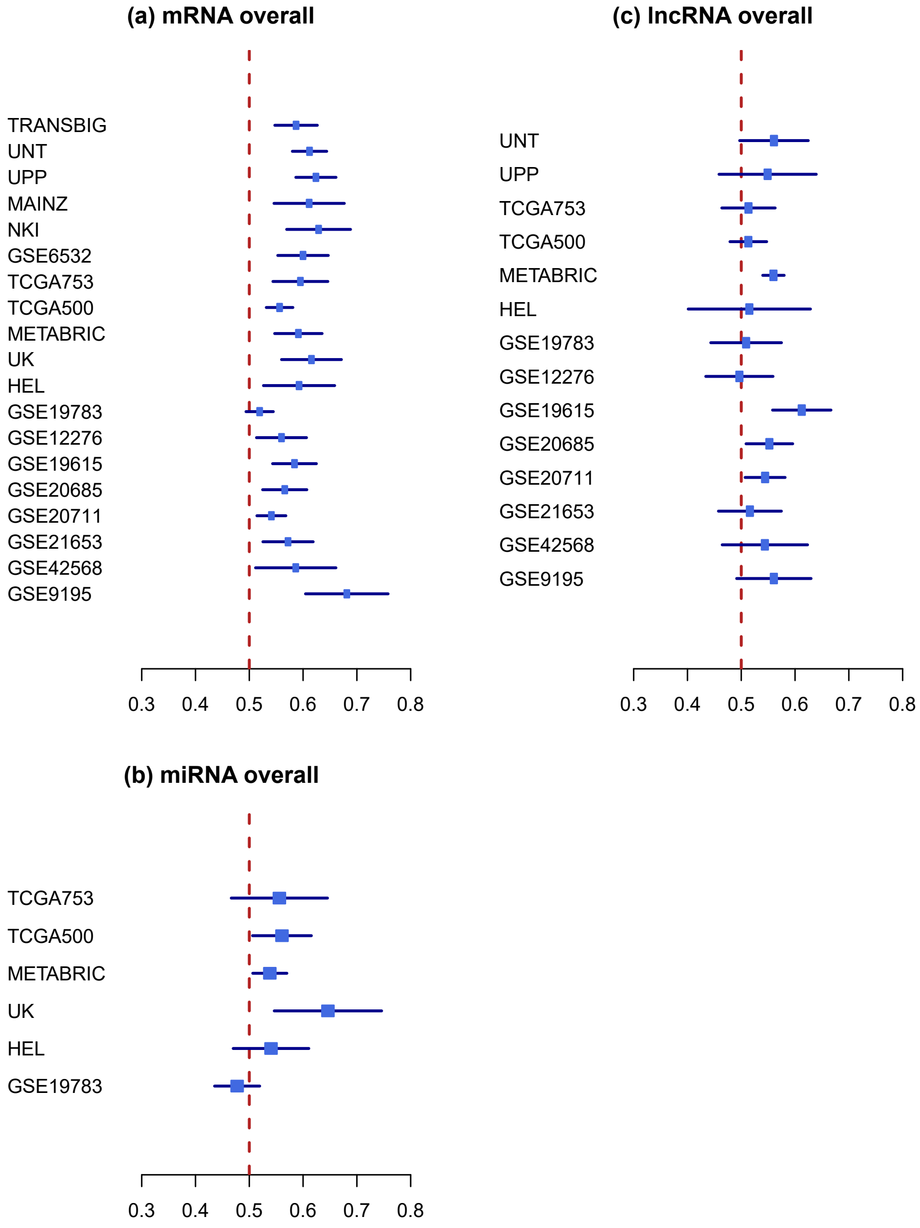

Figure S6: **Forest plots of mean C-indices of different data types.** (a). The performance on the mRNA data. (b). The performance on the miRNA data. (c). The performance on the lncRNA data.

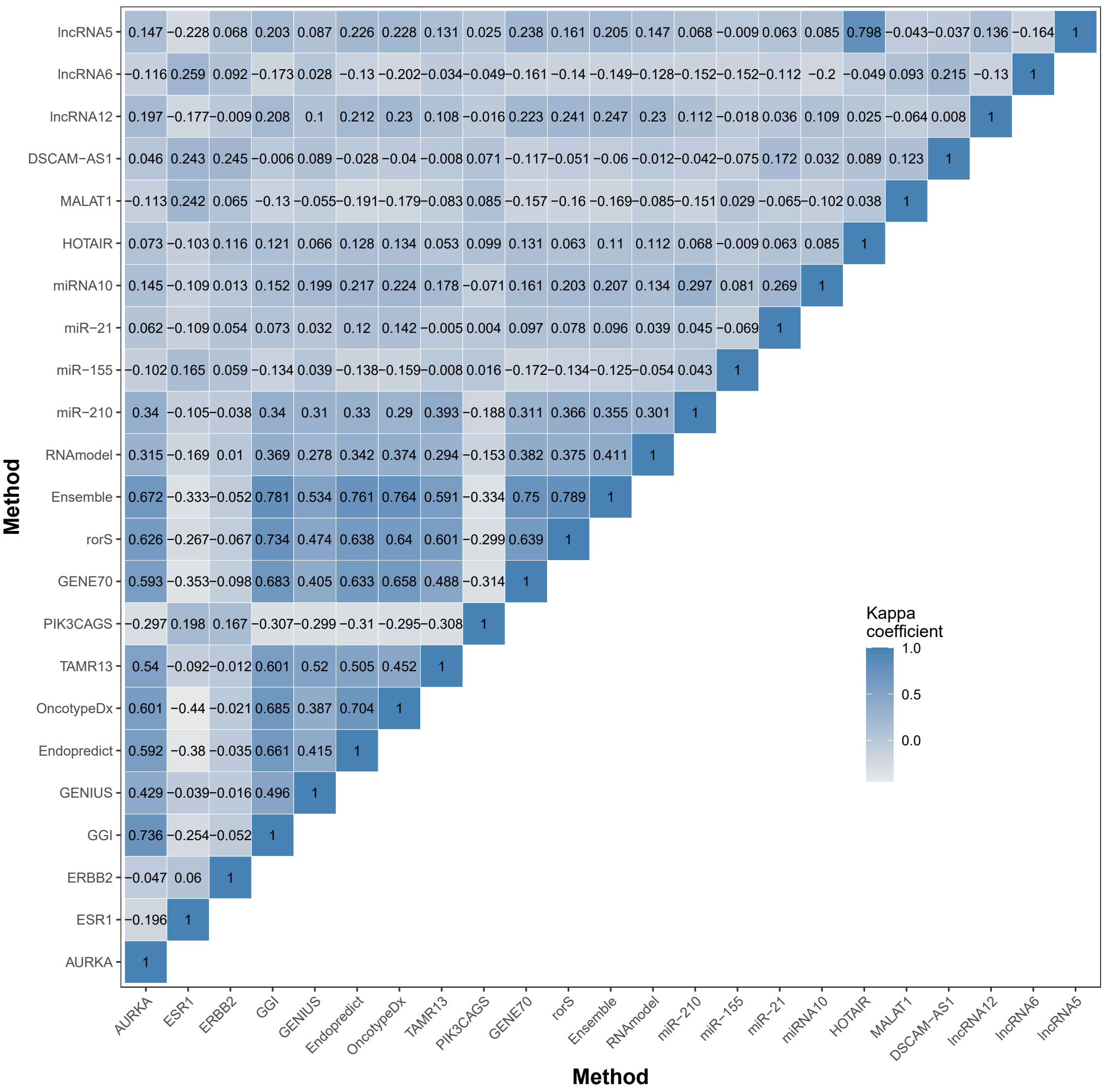

Figure S7: **The Kappa coefficients between different methods.** Both the x-axis and y-axis are methods.

Computational Methods for Breast Cancer Subtyping

Consensus clustering (CC) CC [1] is a framework to obtain the consensus clusters over multiple runs of a clustering algorithm on a subset of samples. For each clustering result, a binary similarity matrix is constructed from the corresponding sample labels: if two samples belong to the same cluster, their similarity is 1, otherwise, the similarity is 0. A consensus matrix is calculated by averaging all similarity matrices of multiple runs of the clustering algorithm. The resulting consensus matrix is clustered using a clustering algorithm, and the optimal number of clusters can be identified by the maximum area under the consensus distribution of the consensus matrix. The TCGA consortium uses CC as a benchmark algorithm to deal with the genomic, epigenomic, transcriptomic and proteomic data for cancer subtypes [2].

Consensus non-negative matrix factorization (CNMF) CNMF [3] combines the non-negative matrix factorization (NMF) method with the consensus clustering framework. The NMF method is used to reducing the dimension of expression data from thousands of genes to few metagenes. However, NMF is sensitive to the random initial conditions and may not converge to the same result for different runs. So consensus clustering is used to obtain the consensus matrix over many clustering runs by hierarchical clustering method based on the metagenes. Similar to CC, CNMF can obtain consensus clusters.

PAM50 PAM50 [4] is a gene-based method to classify samples into five subtypes: Basal, Luminal A, Luminal B, Her2-enriched and Normal-like. PAM50 constructs a centroid-based prediction model by using the Prediction Analysis of Microarray (PAM) algorithm [5] on 50 gene signatures. The 50 gene signatures are selected by two main steps. First, the hierarchical clustering method [6] is used to select informative genes for the prototype samples. Then, a total of 50 genes in the informative gene sets (10 genes for each subtype) are selected based on their contributions in terms of distinguishing the five intrinsic breast cancer subtypes.

Integrative clustering (iCluster) iCluster [7] is an integrative clustering framework simultaneously and flexibly analyzing multiple genomic data sources. Firstly, the Principal Component Analysis (PCA) method [8] and a lasso-type regularization method [9] are used for dimension reduction within data sources and between data sources, respectively. After dimension reduction, joint Gaussian latent variables are extracted from multiple data sources. Then, a K-Means approach is used to obtain the latent tumour subtypes on the joint Gaussian latent variables.

IntClust IntClust [10, 11] is a integrative method to classify samples to ten breast cancer subtypes. IntClust applies iCluster on a matched mRNA-CNV breast cancer dataset with 997 samples and identifies ten breast cancer subtypes (so-called integrative subtypes). Similar to PAM50, IntClust builds three centroid-based prediction models based on 612 *cis* eQTLs gene drivers by using PAM. New patients in the validation set (983 samples) [10] and external independent set (7544 samples) [11] are classified by three versions of PAM: copy number-based classifier, expression-based classifier and combined classifier. The results show that the integrative subtypes by the combined classifier are significantly associated with relapse-free survival and reveal distinct patterns of chemosensitivity.

Similarity Network Fusion (SNF) SNF [12] is a method for clustering multi-omic data. SNF transfers each omic data to a patient-patient similarity network. The nodes represent patients and edges are patients' pairwise similarities. A nonlinear similarity information propagation method is used to calculate a consensus network from the patients' similarity networks. In the consensus network, the edges caused by noise or bias will be degenerate or even disappear, while true similarities supported by most networks are retained. The subtypes are obtained by the spectral clustering algorithm [13] based on the consensus network.

SNF-CC SNF-CC [14] combines SNF and CC to take the advantages of both for cancer subtype identification.

Weighted SNF (WSNF) WSNF [15] calculates patients' pairwise similarities with weighted gene and miRNA expression data before using the SNF method. The weight of a gene/miRNA is a linear combination of a network-based ranking and expression variation of the gene/miRNA. In order to rank genes/miRNAs based on molecular network, miRNA-Transcription Factor (TF)-mRNA regulatory network is constructed from different types of interactions, including miRNA-mRNA, miRNA-TF, TF-miRNA, TF-mRNA, TF-TF and mRNA-mRNA. The median absolute deviation (MAD) method is used to compute the expression variation of a gene/miRNA. The combined gene/miRNA weight benefits both from the prior knowledge of the regulatory network and gene expression variation pattern.

NEighborhood based Multi-Omics clustering (NEMO) NEMO [16] is a similarity-based multi-omic clustering method based on the radial basis of function kernel [17] and spectral clustering method [18]. Different from other multi-omic clustering methods, NEMO can apply to partial data that some omics may not be measured for some patients. NEMO is simple, and faster than other multi-omic clustering methods but achieves comparable performance with others.

Cancer Integration via Multikernel Learning (CIMLR) CIMLR [19] calculates the similarity between patients in multi-omic data by combining a set of Gaussian kernels for each single-omic data. To represent the different and complementary information of a data, a set of 55 gaussian kernels are constructed with different variance for each data type independently. Finally, all the hyperparameters are optimized in one loss function for all the omic data together and one similarity matrix will be learned from the multi-kernels based on all the omic data. The similarity matrix can be used in the K-means clustering method. CIMLR is successfully applied to 36 cancer types on four omics data, and the results show that the method can recover known as well as novel subtypes.

Perturbation clustering for data INtegration and disease Subtyping (PINS) PINS [20] initially clusters patients based on each omic data separately using perturbation clustering and outputs the optimal number of clusters k_i, original connectivity matrix C_i and the perturbed connectivity matrix A_i, where i is the index of ith omic data. The C_i matrices are averaged to obtain the original similarity matrix S_C, where $S_C(i,j) = 0$ if patients i and j are never clustered together in all the omic data, otherwise $S_C(i,j) = 1$. Agglomerative hierarchical clustering (HC), PAM [5], and dynamic tree cut [21] are then applied on the similarity matrix S_C or distance matrix $1 - S_C$, and select the clusters that are highly agreement with the clusters on each omic data. Unlike the previously presented multi-omic clustering methods, PINS further splits the initial clusters into small clusters by checking the consistency between patients within each group or testing whether the cluster is balanced.

An integrative approach for disease subtype classification based on non-negative matrix factorization (intNMF) intNMF [22] is an integrative clustering method for multi-omics data based on a generalized NMF. intNMF factorizes each omic matrix $X^i_{n\times p}$ into two matrices with positive entries, i.e. $X^i_{n\times p} \sim W_{n\times k}H^i_{k\times p}$, where n and p are the sample size and the number of features in the ith omic data, k is the number of clusters. The factor matrix W holds the common clustering information based on multi-omics data, and $H^i_{k\times p}$ represents the omic specific matrix of coefficient vectors. The matrices W and $H^i_{k\times p}$ are computed by a faster non-negativity constrained alternating least square algorithm [23]. Each sample j is assigned to the cluster based on the highest entry in the jth row of W.

Computational Methods for Breast Cancer Prognosis

Gene70 Gene70 [24] is developed to forecast the risk of breast cancer distant metastases of primary invasive breast cancer patients who are diagnosed with lymph node-negative, stage 12, and tumour size smaller than 5.0 cm. It first uses an unsupervised clustering method to cluster 98 tumours into two groups and retain about 5000 genes that are important to the clustering. Then, Pearson coefficients are calculated between genes and prognostic categories (metastasis and no-metastasis). 231 genes are kept as correlated (Pearson coefficient is larger

than 0.3) or anti-correlated genes (Pearson coefficient is less than -0.3). Finally, 70 prognostic gene signatures are inferred by a supervised classification method. These gene signatures are enriched in cell proliferation, invasion, metastasis, stromal integrity and angiogenesis. The risk score is calculated by using a correlation coefficient between the expression profiles of the 70 genes in a patient and the average good prognosis profile (average profile of patients who are free of disease at least 5 years). Gene70 aims to reduce the rates of over-treatment and under-treatment by identifying high-risk patients who may benefit from adjuvant therapy and those that may safely forgo chemotherapy, respectively. Subsequent studies extended Gene70 to lymph node-positive disease [25] , HER2+ breast cancer [26], ER+ and HER2- early breast cancer [27]. However, it provides a few prognostic information to clinicopathologic risk assessment for ER-patients [28].

OncotypeDX OncotypeDX [29] is a 21-gene based method and the 21 gene signatures come from an RT-PCR assay of formalin-fixed paraffin-embedded (FFPE) tissues. This method firstly selects 250 candidate genes from published databases or literature. After analyzing the relation between 250 candidate genes and the distant recurrence in 447 patients (node(-), ER+, including tamoxifen monotherapy), 16 cancer-related genes and 5 reference genes are selected to build a linear prediction model. The coefficients of the 21 genes in the prediction model are determined by the Cox regression models from the previous three studies. The model outputs a Recurrence Score ($RS \subset [0, 100]$) for a new patient. The recurrence score indicates the likelihood of distant recurrence and chemotherapy benefits for a patient, and the value of RS can be divided into three levels: $RS < 18$ (low-risk), $RS \subset [18 - 31]$ (intermediate-risk), $RS > 31$ (high-risk).

Gene expression Grade Index (GGI) GGI [30] selects 97 gene signatures according to their differentially expressed between histologic grade 1 and 3 frozen-fresh tumours. Firstly, the grade 2 tumours need to be adjusted into a grade 1 like group or a grade 3 like group that have similar clinical behaviour to the samples in the grade 1 group or the grade 3 group, respectively. Then, 97 grade-associated gene signatures are found to be associated with grade 1 or/and grade 3. The difference between the expression profiles of the grade 1 and 3 associated genes is used to indicate the gene expression grade index of a patient. A high gene expression grade index corresponds to a high risk of relapse for a patient and vice versa. There is a related method PCR-GGI [31] can work on qRT-PCR assay instead of the microarray. PCR-GGI has 8 gene signatures which are sufficient to recapitulate the performance of the original GGI.

rorS RorS [4] is a risk predictor based on the breast cancer subtypes identified by PAM50. The method is trained by a Cox model with a Ridge regression on node-negative, untreated samples. The risk score of a new patient can be computed by $rorS = 0.05 \cdot basal + 0.12 \cdot HER2 - 0.34 \cdot LumA + 0.23 \cdot LumB$. If combined the coefficients from the subtypes and tumour size, the result is superior to either clinical information alone or subtypes alone.

Tamoxifen resistance signature (TAMR13) TAMR13 [32] extracts tamoxifen resistance signatures based on the treatment response to tamoxifen since $30 - 40\%$ of ER+ breast cancer patients developed distant metastases and died despite the treatment. A hierarchical clustering method is used to cluster genes to 110 clusters. The cluster centroid (denoted as $pclust$) is ranked by the likelihood ratio statistic of the univariate Cox model. To maximize the stability criterion, 13 cluster centroids are selected as the tamoxifen resistance signatures. The TAMR13 risk score is calculated by $\sum_{j=1}^{13} \beta_j \cdot pclust_j$, where β_j is the coefficient of $pclust_j$ by the univariate Cox model. Results on ER+ cohort show that the tamoxifen resistance signatures might be significantly responsible for clinical outcomes and treatment response to tamoxifen.

Gene Expression progNostic Index Using Subtypes (GENIUS) GENIUS [33] utilizes a fuzzy computational approach and a divide-conquer strategy for breast cancer prognosis. Similar to rorS, GENIUS takes into account the presence of molecular subtypes for the identification of prognostic gene signatures. A stability-based feature ranking method (based on weighted concordance index) is used to identify the informative genes in each molecular subtype (ER-/HER2-, HER2+ or ER+/HER2-). Then, a subtype risk score is computed by a fuzzy computational approach for each subtype. Finally, the GENIUS risk score is defined as the combination of weighted (determined by a Cox model) subtype risk scores.

PIK3CAGS PIK3CAGS [34] calculates risk scores based PIK3CA mutation-associated gene signatures. It has been found that the PIK3CA mutations are associated with an optimistic expectation of tamoxifen monotherapy. PIK3CA mutation-associated gene signatures are selected because of their ability to predict PIK3CA mutation status of a sample. The PIK3CAGS risk score is the sum of differences between the expression levels of up-regulated genes in the mutated tumours and those in the wild-type tumours. However, the prognosis value of PIK3CAGS is limited to ER+/HER2- breast cancer cohort. The performance of PIK3CAGS in other subtypes of breast cancer cohort needs to be evaluated further.

EndoPredict EndoPredict [35] is applied to calculate the risk of distant recurrence for patients diagnosed with ER+, HER2- subtype and treated with adjuvant endocrine therapy but without reliance on clinicopathological. The EndoPredict signatures contain 8 cancer-related genes of interest and 3 normalization genes. The continuous EndoPredict risk score is calculated by a linear model based on EndoPredict signatures, node status, and tumour size. The Cox regression method is used in both gene ranking and linear coefficients estimation. Compared to previous methods, EndoPredict results in a significant improvement of the prognostic performance on the early-stage breast cancer dataset.

Ensemble Ensemble calculates the average of predicted risk scores from 5 methods, GENIUS, EndoPredict, OncotypeDX, GENE70, and rorS. We chose these five methods because of their good performance.

Single-signature methods Single-signature models contain 3 gene-based methods (AURKA, ESR1, and ERBB2), 3 miRNA-based methods (miR-21, miR-155, and miR-210) and 3 lncRNA-based methods (MALAT1, HOTAIR, and DSCAM-AS1). AURKA, ESR1, and ERBB2 are selected to represent three biological processes (proliferation, ER signalling and HER2 signalling) in breast cancer, respectively [36]. Compare to IHC4 [37], AURKA is superior to Ki67 as a prognostic indicator of survival in ER+ breast cancer [38]. Three selected miRNAs have been proved to associate with clinical outcomes of breast cancer patients. For example, the up-regulation of miR-21 is associated with poor prognosis and lymph node metastasis [39, 40, 41]. MiR-155 is over-expressed in tumour tissues and correlated with better outcomes [42]. Over-expression of miR-210 is associated with poor survival outcome and could be used as an independent prognostic marker [43, 44]. LncRNAs could dysregulate cancer pathways, oncogenes and tumour suppressors through their effects on miRNAs, genes, transcription factors and RNA-binding proteins. The two most studied lncRNAs are MALAT1 and HOTAIR. High expression of MALAT1 in breast cancer is associated with poor relapse-free survival in ER+ patients [45]. MALAT1 promotes proliferation and invasion abilities of cancer cells in TNBC and HER2 patients through XBP1-HIF-1α pathway and Her-2 pathway [46]. HOTAIR competes with BRCA1, which is a critical tumour suppressor in breast cancer and a critical regulator of genes involved in EMT [47]. DSCAM-AS1 implicates carcinogenesis, tumour growth and treatment resistance of breast cancer [48]. A single-signature based method build a univariate cox model based on a single signature (e.g. AURKA), survival time, and event status. Based on these single-signature methods, we can provide insight into the potential clinical implication of a single signature in breast cancer prognosis.

RNAmodel RNAmodel [49] uses two steps to select 30 mRNAs and 7 miRNAs assisted by DNA methylation and somatic mutations data. Firstly, univariate survival analysis conducts to select initial mRNA/miRNA set with the high relationship between clinical parameters and outcomes in the TCGA invasive ductal carcinomas (IDC) cohort. The criterion is that the RNAs should have significant test results in at least two subclasses by the hazard ratio (HR) and the Log-rank tests. In addition, the mRNAs should have at least 2 mutations in the COSMIC database (`https://cancer.sanger.ac.uk/cosmic`) and be associate with overall survival time on the methylated CpG sites. A further prognostic RNA performance (the maximal area under curve AUC for each major breast subtype) is assessed by the supervised principal component method [50] and subtype-directed RNA wrapper selected method. Finally, an mRNA/miRNA linear risk prediction model is conducted to predict the risk score of new coming patients.

miRNA10 miRNA10 [51] uses a two-step Cox analysis to select miRNA signatures on the UK dataset. Firstly, miRNAs associated with Distant Relapse Free Survival time are selected by L_1 and L_2 penalized regression on the miRNA data. Then, to make sure that the miRNA signatures are independently from other factors to breast cancer prognosis, a Cox model is carried on all miRNAs, genes and clinical covariates. This procedure identifies 10 prognostic miRNAs that enrich in key biological processes including proliferation, ESR1 and HER2 signalling, hypoxia, invasion, immune response, stem cell, and apoptosis.

lncRNA12 lncRNA12 [52] builds a BCSigLnc-12 risk score model to calculated risk scores of patients and classified them into the high-risk group and the low-risk group with significantly different recurrence-free survival pattern. The risk scores are calculated by a linear combination of expression profiles of 12 lncRNAs with the estimated regression coefficients.

LncRNA6 LncRNA6 [53] is based on a 6-lncRNA linear risk score model to predict the overall survival rate in ER+ breast cancer patients. Six independent prognostic lncRNAs are selected by using a multivariate Cox regression model to obtain their weights in predicting overall survival. Then similar to lncRNA12, a linear model is built to calculate the risk scores of patients.

LncRNA5 LncRNA5 [54] identifies lncRNA signatures based on two criteria: survival-related and breast cancer related functions. Only 5 lncRNAs (RP11524D16-A.3, HOTAIR, AL645608.1, TSPOAP1-AS1 and RP11-13L2.4) are left after univariate Cox proportional hazards regression analysis and functions investigation. The experiment showed that the five-lncRNA signature was significantly associated with disease-free survival and independent of subtypes and adjuvant treatments factors.

Performance evaluation

The Silhouette width The Silhouette width [55] measures how similar a sample is to its subtype compared to other subtypes. The Silhouette width calculates by any distance metric or similarity metric and ranges from -1 to 1. A high value indicates that the sample is well matched to its subtype and poorly matched to other subtypes. Let $a(x_i)$ be the average distance of sample x_i to other samples of its cluster, $b(x_i)$ be the average distance of sample x_i to all the other samples of the other cluster. The Silhouette width sw_i and the average Silhouette width (called the Silhouette score) SC are defined as Equation (1).

$$\begin{aligned} sw_i &= \frac{b(x_i) - a(x_i)}{max\,\{a(x_i), b(x_i)\}} \\ SC &= \frac{1}{n}\sum_{i}^{n} sw_i \end{aligned} \quad (1)$$

Where n is the number of samples. If an algorithm provides a similarity matrix S, we transfer the similarity matrix to the distance matrix. This will automatically be transferred by our R package. The Silhouette plot displays the Silhouette width for each sample and the summarized information, including the number of clusters, average Silhouette widths in each subtype and the Silhouette score.

Concordance index C-index [56] is defined as the proportion of all comparable pairs in which the predictions and outcomes are concordant. For two comparable patients, the concordance between predictions and outcomes means that the patient with the higher risk prediction experienced an event (e.g. death) before the one with the lower risk prediction. Without loss of generality, we suppose patient i has the longer observed survival time t_i and patient j has the shorter one t_j. And patient j experimented with an event during the follow-up time. Let δ_j be the censoring status. An event (e.g. death) is developed within the study period when $\delta_j = 1$. C-index can be computed with the following equation:

$$\text{C-index} = \frac{\sum_{i,j} I(t_i > t_j) I(r_i < r_j) \delta_j}{\sum_{i,j} I(t_i > t_j) \delta_j} \tag{2}$$

where *I()* is an indication function. C-index ranges from 0 to 1. If the C-index of a method equals 0.5, that means this method is no better than a random guess model. C-index=1 means that the predictions and outcomes are perfect concordant. The C-indices of poor methods may less than 0.5. The range of values C-index can be divided into four levels that describe the goodness of fit for a cancer prognosis method.

- Poor model: [0,0.5]
- Intermediate model: (0.5, 0.7]
- Good model: (0.7, 0.8]
- Strong model: (0.8, 1.0]

The Kaplan-Meier method Kaplan-Meier method is a non-parametric statistic used to estimate the survival function from clinical outcome data. The survival function stands for the probability of patients living for a certain amount of event times. The survival function $S(t \geqslant t_j)$ can be estimated by

$$\hat{S}(t) = \prod_{i:t_i \leq t} \left[1 - \frac{d_i}{n_i}\right] \tag{3}$$

Where t_i is the ith distinct event time when at least one event happened (e.g. death). d_i is the number of events that happened at time t_i. n_i is the number of individuals who are censored at time t_i or have not experienced an event before time t_i. In the KM curve plot, the Y-axis is the probability of surviving in a given length of time and the X-axis is serial distinct event times. The KM curves for different groups should have different characteristics and not overlap in good methods.

The Log-rank test The Log-rank test assesses whether the survival curves estimated for two or more groups are identical or not. The null hypothesis H_0 is that there is no difference between the survival distribution at any time point. The p-value of the Log-rank rest for a method is less than 0.05, thus the survival curves are statistically significantly different.

The Cohen's kappa coefficient The Cohen's kappa coefficient measures the agreement for two predictors with the aim of evaluating the inter-predictors reliability. The definition of Cohen's kappa coefficient (denoted as k) is:

$$k = \frac{p_o - p_e}{1 - p_e} \tag{4}$$

Where p_o is the observed agreement and p_e is the chance agreement. We define the p_o and p_e as follows

$$\begin{aligned} p_o &= \frac{a+d}{n} \\ p_e &= \frac{(a+b)(a+c)+(c+d)(b+d)}{n^2} \end{aligned} \tag{5}$$

Where n is the number of samples, a is the number of samples which both predictors assign to the high-risk group, b is the number of samples which the first predictor assigns to the high-risk group but the second predictor assigns to the low-risk group, c is the number of samples which the first predictor assigns to the low-risk group but the second predictor assigns to the high-risk group, and d is the number of samples which both predictors assign to the low-risk group.

The range of values k is divided into six levels that describe the concordance between two methods.

- Not effective agreement: less than 0
- Slight agreement: [0, 0.20]
- Fair agreement: (0.20, 0.40]
- Moderate agreement: (0.40, 0.60]
- Substantial agreement: (0.60, 0.80]
- Almost perfect agreement: (0.80, 1.00]

References

[1] Monti S, Tamayo P, Mesirov J, Golub T. Consensus clustering: a resampling-based method for class discovery and visualization of gene expression microarray data. Machine learning. 2003;52(1-2):91–118.

[2] Berger AC, Korkut A, Kanchi RS, Hegde AM, Lenoir W, Liu W, et al. A comprehensive Pan-Cancer molecular study of gynecologic and breast cancers. Cancer Cell. 2018;33(4):690–705.

[3] Brunet JP, Tamayo P, Golub TR, Mesirov JP. Metagenes and molecular pattern discovery using matrix factorization. Proceedings of the National Academy of Sciences. 2004;101(12):4164–4169.

[4] Parker JS, Mullins M, Cheang MC, Leung S, Voduc D, Vickery T, et al. Supervised risk predictor of breast cancer based on intrinsic subtypes. Journal of clinical oncology. 2009;27(8):1160.

[5] Tibshirani R, Hastie T, Narasimhan B, Chu G. Diagnosis of multiple cancer types by shrunken centroids of gene expression. Proceedings of the National Academy of Sciences. 2002;99(10):6567–6572.

[6] Eisen MB, Spellman PT, Brown PO, Botstein D. Cluster analysis and display of genome-wide expression patterns. Proceedings of the National Academy of Sciences. 1998;95(25):14863–14868.

[7] Shen R, Olshen AB, Ladanyi M. Integrative clustering of multiple genomic data types using a joint latent variable model with application to breast and lung cancer subtype analysis. Bioinformatics. 2009;25(22):2906–2912.

[8] Tipping ME, Bishop CM. Probabilistic principal component analysis. Journal of the Royal Statistical Society: Series B (Statistical Methodology). 1999;61(3):611–622.

[9] Tibshirani R. Regression shrinkage and selection via the lasso. Journal of the Royal Statistical Society Series B (Methodological). 1996;58(1):267–288.

[10] Curtis C, Shah SP, Chin SF, Turashvili G, Rueda OM, Dunning MJ, et al. The genomic and transcriptomic architecture of 2,000 breast tumours reveals novel subgroups. Nature. 2012;486(7403):346.

[11] Ali HR, Rueda OM, Chin SF, Curtis C, Dunning MJ, Aparicio SA, et al. Genome-driven integrated classification of breast cancer validated in over 7,500 samples. Genome biology. 2014;15(8):431.

[12] Wang B, Mezlini AM, Demir F, Fiume M, Tu Z, Brudno M, et al. Similarity network fusion for aggregating data types on a genomic scale. Nature methods. 2014;11(3):333.

[13] Bach F, Jordan M. Learning spectral clustering. Advances in neural information processing systems. 2004;16(2):305–312.

[14] Xu T, Le TD, Liu L, Su N, Wang R, Sun B, et al. CancerSubtypes: an R/Bioconductor package for molecular cancer subtype identification, validation and visualization. Bioinformatics. 2017;33(19):3131–3133.

[15] Xu T, Le TD, Liu L, Wang R, Sun B, Li J. Identifying cancer subtypes from mirna-tf-mrna regulatory networks and expression data. PloS one. 2016;11(4):e0152792.

[16] Rappoport N, Shamir R. NEMO: cancer subtyping by integration of partial multi-omic data. Bioinformatics. 2019;35(18):3348–3356.

[17] Buhmann MD. Radial basis functions: theory and implementations. vol. 12. Cambridge university press; 2003.

[18] Von Luxburg U. A tutorial on spectral clustering. Statistics and computing. 2007;17(4):395–416.

[19] Ramazzotti D, Lal A, Wang B, Batzoglou S, Sidow A. Multi-omic tumor data reveal diversity of molecular mechanisms that correlate with survival. Nature communications. 2018;9(1):4453.

[20] Nguyen T, Tagett R, Diaz D, Draghici S. A novel approach for data integration and disease subtyping. Genome research. 2017;27(12):2025–2039.

[21] Langfelder P, Zhang B, Horvath S. Defining clusters from a hierarchical cluster tree: the Dynamic Tree Cut package for R. Bioinformatics. 2008;24(5):719–720.

[22] Chalise P, Fridley BL. Integrative clustering of multi-level 'omic data based on non-negative matrix factorization algorithm. PloS one. 2017;12(5):e0176278.

[23] Van Benthem MH, Keenan MR. Fast algorithm for the solution of large-scale non-negativity-constrained least squares problems. Journal of Chemometrics: A Journal of the Chemometrics Society. 2004;18(10):441–450.

[24] Van't Veer LJ, Dai H, Van De Vijver MJ, He YD, Hart AA, Mao M, et al. Gene expression profiling predicts clinical outcome of breast cancer. Nature. 2002;415(6871):530.

[25] Mook S, Schmidt MK, Viale G, Pruneri G, Eekhout I, Floore A, et al. The 70-gene prognosis-signature predicts disease outcome in breast cancer patients with 1–3 positive lymph nodes in an independent validation study. Breast cancer research and treatment. 2009;116(2):295–302.

[26] Knauer M, Cardoso F, Wesseling J, Bedard PL, Linn S, Rutgers E, et al. Identification of a low-risk subgroup of HER-2-positive breast cancer by the 70-gene prognosis signature. British journal of cancer. 2010;103(12):1788.

[27] Exner R, Bago-Horvath Z, Bartsch R, Mittlboeck M, Retel V, Fitzal F, et al. The multigene signature MammaPrint impacts on multidisciplinary team decisions in ER+, HER2- early breast cancer. British journal of cancer. 2014;111(5):837.

[28] Buyse M, Loi S, Van't Veer L, Viale G, Delorenzi M, Glas AM, et al. Validation and clinical utility of a 70-gene prognostic signature for women with node-negative breast cancer. Journal of the National Cancer Institute. 2006;98(17):1183–1192.

[29] Paik S, Shak S, Tang G, Kim C, Baker J, Cronin M, et al. A multigene assay to predict recurrence of tamoxifen-treated, node-negative breast cancer. New England Journal of Medicine. 2004;351(27):2817–2826.

[30] Sotiriou C, Wirapati P, Loi S, Harris A, Fox S, Smeds J, et al. Gene expression profiling in breast cancer: understanding the molecular basis of histologic grade to improve prognosis. Journal of the National Cancer Institute. 2006;98(4):262–272.

[31] Toussaint J, Sieuwerts AM, Haibe-Kains B, Desmedt C, Rouas G, Harris AL, et al. Improvement of the clinical applicability of the Genomic Grade Index through a qRT-PCR test performed on frozen and formalin-fixed paraffin-embedded tissues. BMC genomics. 2009;10(1):424.

[32] Loi S, Haibe-Kains B, Desmedt C, Wirapati P, Lallemand F, Tutt AM, et al. Predicting prognosis using molecular profiling in estrogen receptor-positive breast cancer treated with tamoxifen. BMC genomics. 2008;9(1):239.

[33] Haibe-Kains B, Desmedt C, Rothé F, Piccart M, Sotiriou C, Bontempi G. A fuzzy gene expression-based computational approach improves breast cancer prognostication. Genome biology. 2010;11(2):R18.

[34] Loi S, Haibe-Kains B, Majjaj S, Lallemand F, Durbecq V, Larsimont D, et al. PIK3CA mutations associated with gene signature of low mTORC1 signaling and better outcomes in estrogen receptor–positive breast cancer. Proceedings of the National Academy of Sciences. 2010;107(22):10208–10213.

[35] Filipits M, Rudas M, Jakesz R, Dubsky P, Fitzal F, Singer CF, et al. A new molecular predictor of distant recurrence in ER-positive, HER2-negative breast cancer adds independent information to conventional clinical risk factors. Clinical Cancer Research. 2011;17(18):6012–6020.

[36] Desmedt C, Haibe-Kains B, Wirapati P, Buyse M, Larsimont D, Bontempi G, et al. Biological processes associated with breast cancer clinical outcome depend on the molecular subtypes. Clinical cancer research. 2008;14(16):5158–5165.

[37] Cuzick J, Dowsett M, Pineda S, et al. Prognostic value of a combined estrogen receptor, progesterone receptor, Ki-67, and human epidermal growth factor receptor 2 immunohistochemical score and comparison with the genomic health recurrence score in early breast cancer. Journal of Clinical Oncology. 2011;27(32):4273.

[38] Ali H, Dawson S, Blows F, Provenzano E, Pharoah P, Caldas C. Aurora kinase A outperforms Ki67 as a prognostic marker in ER-positive breast cancer. British journal of cancer. 2012;106(11):1798.

[39] Lee JA, Lee HY, Lee ES, Kim I, Bae JW. Prognostic implications of microRNA-21 overexpression in invasive ductal carcinomas of the breast. Journal of breast cancer. 2011;14(4):269–275.

[40] Yan LX, Huang XF, Shao Q, Huang MY, Deng L, Wu QL, et al. MicroRNA miR-21 overexpression in human breast cancer is associated with advanced clinical stage, lymph node metastasis and patient poor prognosis. Rna. 2008;14(11):2348–2360.

[41] Markou A, Yousef GM, Stathopoulos E, Georgoulias V, Lianidou E. Prognostic significance of metastasis-related microRNAs in early breast cancer patients with a long follow-up. Clinical chemistry. 2014;60(1):197–205.

[42] Gasparini P, Cascione L, Fassan M, Lovat F, Guler G, Balci S, et al. microRNA expression profiling identifies a four microRNA signature as a novel diagnostic and prognostic biomarker in triple negative breast cancers. Oncotarget. 2014;5(5):1174.

[43] Camps C, Buffa FM, Colella S, Moore J, Sotiriou C, Sheldon H, et al. hsa-miR-210 Is induced by hypoxia and is an independent prognostic factor in breast cancer. Clinical cancer research. 2008;14(5):1340–1348.

[44] Wang J, Zhao J, Shi M, Ding Y, Sun H, Yuan F, et al. Elevated expression of miR-210 predicts poor survival of cancer patients: a systematic review and meta-analysis. PloS one. 2014;9(2):e89223.

[45] Wang Z, Katsaros D, Biglia N, Shen Y, Fu Y, Loo LW, et al. High expression of long non-coding RNA MALAT1 in breast cancer is associated with poor relapse-free survival. Breast cancer research and treatment. 2018;171(2):261–271.

[46] Xiping Z, Bo C, Shifeng Y, Feijiang Y, Hongjian Y, Qihui C, et al. Roles of MALAT1 in development and migration of triple negative and Her-2 positive breast cancer. Oncotarget. 2018;9(2):2255.

[47] Pawłowska E, Szczepanska J, Blasiak J. The Long Noncoding RNA HOTAIR in Breast Cancer: Does Autophagy Play a Role? International journal of molecular sciences. 2017;18(11):2317.

[48] Niknafs YS, Han S, Ma T, Speers C, Zhang C, Wilder-Romans K, et al. The lncRNA landscape of breast cancer reveals a role for DSCAM-AS1 in breast cancer progression. Nature communications. 2016;7:12791.

[49] Volinia S, Croce CM. Prognostic microRNA/mRNA signature from the integrated analysis of patients with invasive breast cancer. Proceedings of the National Academy of Sciences. 2013;110(18):7413–7417.

[50] Bair E, Tibshirani R. Semi-supervised methods to predict patient survival from gene expression data. PLoS biology. 2004;2(4):e108.

[51] Buffa FM, Camps C, Winchester L, Snell CE, Gee HE, Sheldon H, et al. microRNA-associated progression pathways and potential therapeutic targets identified by integrated mRNA and microRNA expression profiling in breast cancer. Cancer research. 2011;71(17):5635–5645.

[52] Zhou M, Zhong L, Xu W, Sun Y, Zhang Z, Zhao H, et al. Discovery of potential prognostic long non-coding RNA biomarkers for predicting the risk of tumor recurrence of breast cancer patients. Scientific reports. 2016;6(1):1–11.

[53] Zhong L, Lou G, Zhou X, Qin Y, Liu L, Jiang W. A six-long non-coding RNAs signature as a potential prognostic marker for survival prediction of ER-positive breast cancer patients. Oncotarget. 2017;8(40):67861.

[54] Li J, Wang W, Xia P, Wan L, Zhang L, Yu L, et al. Identification of a five-lncRNA signature for predicting the risk of tumor recurrence in patients with breast cancer. International journal of cancer. 2018;143(9):2150–2160.

[55] Rousseeuw PJ. Silhouettes: a graphical aid to the interpretation and validation of cluster analysis. Journal of computational and applied mathematics. 1987;20:53–65.

[56] Harrell FE, Lee KL, Mark DB. Multivariable prognostic models: issues in developing models, evaluating assumptions and adequacy, and measuring and reducing errors. Statistics in medicine. 1996;15(4):361–387.
